# Synergistic activation of the NEU4 promoter by p73 and AP2 in colon cancer cells

**DOI:** 10.1038/s41598-018-37521-7

**Published:** 2019-01-30

**Authors:** Bi-He Cai, Po-Han Wu, Chi-Kan Chou, Hsiang-Chi Huang, Chia-Chun Chao, Hsiao-Yu Chung, Hsueh-Yi Lee, Jang-Yi Chen, Reiji Kannagi

**Affiliations:** 1 Institute of Biomedical Sciences, Academia Sinica, Taipei, Taiwan; 2 Department of Biology and Anatomy, National Defense Medical Center, Taipei, Taiwan; 3 Taiwan International Graduate Program in Molecular Medicine, National Yang-Ming University and Academia Sinica, Taipei, Taiwan; 4 Genomics Research Center, Academia Sinica, Taipei, Taiwan

## Abstract

More than 50% of colon cancers bear mutations in p53, one of the most important tumor suppressors, and its family members p63 or p73 are expected to contribute to inhibiting the progression of colon cancers. The AP2 family also acts as a tumor suppressor. Here we found that p73 and AP2 are able to activate NEU4, a neuraminidase gene, which removes the terminal sialic acid residues from cancer-associated glycans. Under serum starvation, NEU4 was up-regulated and one of the NEU4 target glycans, sialyl Lewis X, was decreased, whereas p73 and AP2 were up-regulated. Sialyl Lewis X levels were not, however, decreased under starvation conditions in p73- or AP2-knockdown cells. p53 and AP2 underwent protein-protein interactions, exerting synergistic effects to activate p21, and interaction of p53 with AP2 was lost in cells expressing the L350P mutation of p53. The homologous residues in p63 and p73 are L423 and L377, respectively. The synergistic effect of p53/p63 with AP2 to activate genes was lost with the L350P/L423P mutation in p53/p63, but p73 bearing the L377P mutation was able to interact with AP2 and exerted its normal synergistic effects. We propose that p73 and AP2 synergistically activate the NEU4 promoter in colon cancer cells.

## Introduction

Glycans play fundamental roles in key pathological steps of tumor development and progression^1^. Sialyl Lewis X and sialyl Lewis A are highly expressed in colon cancer cells^2–5^. The epithelial–mesenchymal transition (EMT) is the process by which cancer stem-like cells are enriched^6,7^. We previously induced EMT in DLD1 and HT29 cells using EGF and bFGF and found that expression of the cancer-associated glycans sialyl Lewis X and sialyl Lewis A is markedly enhanced in EMT-induced cells^4^. NEU4 is a neuraminidase and removes terminal sialic acid residues on cancer-associated glycans such as sialyl Lewis X, sialyl Lewis A and polysialylated NCAM (PSA-NCAM)^8,9^. *NEU4* expression is reduced in colon cancer patients, and its expression may be related to cancer cell apoptosis^10^. EGF can enhance Src signaling^11^, and Src can phosphorylate Wwox at Y33 to enhance Wwox-p73 and Wwox-AP2γ interactions to block p73 and AP2γ activity, respectively^12,13^. As EMT induced by EGF and bFGF represses NEU4 expression, we speculated that p73 and AP2 may be involved in NEU4 regulation.

The AP2 and p53 families are tumor suppressor genes^14–16^. AP2α and AP2γ are reduced in colon cancer patients^17^. AP2α and AP2γ interact with p53^18^. AP2 can act as a co-regulator that binds to the same site as p63 to regulate epidermal differentiation^19^. p53 is a tumor suppressor and can induce cell cycle arrest proteins such as p21 and 14-3-3σ^20,21^. p53 is mutated in >50% of colon cancer patients^22^, and close to 50% of colon cancer cell lines have p53 mutations^23^. A loss-of-function mutation in p53 causes cells to lose their cell cycle check points and cell arrest function and thus leads to their abnormal proliferation^24^. In contrast, p63 and p73, two other members of the p53 family, are rarely mutated in cancer patients^25^. p73 has several isoforms such as its transactivation form (TA) and dominant-negative forms (ΔN and ΔN’)^26^. p63 and p73 have more isoforms than p53, and the dominant-negative isoform ΔNp63α is the major form of p63 in adult cells^27^. Transactivation isoforms TAp63 and TAp73 are expressed in colon cells and play a role in repressing cancer progression^28–30^. Because all the p53 members have a C-terminal tetramerization domain that allows them to form tetramers, the re-activation of endogenous p73 is a good strategy for killing p53-mutated colon cancer cells^31^. The presence of one ΔN isoform of a p53 family member within a tetramer blocks the transactivation function of that tetramer, but three p53 family members within a tetramer must be mutated to block the function of a tetramer^32^. This means that re-activation of >25% of TAp73 relative to the amount of mutated p53 is enough to rescue the tetramer function of p73 to trigger its cell death function.

Here we found that p73 and AP2 could bind and activate the NEU4 promoter in p53-mutated colon cancer cells. Repression of p73 or AP2 reduced NEU4 expression and rescued the starvation-mediated up-regulation of NEU4 and reduction of sialyl Lewis X glycans. As sialyl Lewis X is a major ligand for endothelial selectins and facilitates hematogenous metastasis of cancer cells through mediating the adhesion of cancer cells to vascular endothelial cells^33,34^, reduction of sialyl Lewis X glycans is expected to reduce metastatic activity.

## Results

### NEU4, AP2 and p73 transcript profiles in colon cancer cells

**Figure 1:**
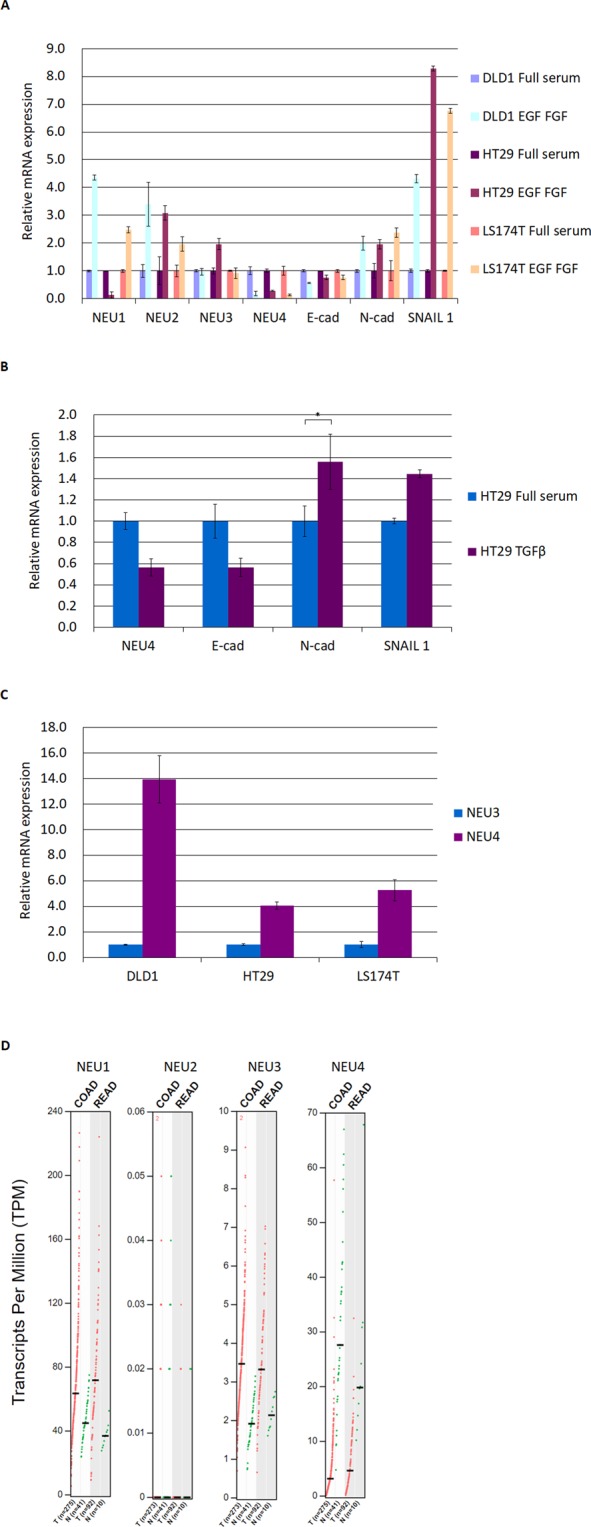
NEU4 expression is decreased upon EMT in colon cancer cells. (**A**) NEU4 levels were reduced, but levels of other sialidases were most increased or no changes upon EMT induced by EGF and bFGF in DLD1, HT29 and LS174T cells. EGF and bFGF treatment reduced the level of the epithelial marker E-cadherin and enhanced mesenchymal markers such as N-cadherin and SNAIL 1. (**B**) NEU4 is decreased upon EMT induced by TGF-β in HT29 cells. (**C**) NEU4 showed much higher expression than NEU3 in DLD1, HT29 and LS174T cells. (**A**–**C**) Results are displayed as mean ± SD, n = 3. (*p < 0.05) (**D**) NEU4 is decreased in colon adenocarcinoma (COAD) and rectum adenocarcinoma (READ), but NEU1 and NEU3 are up-regulated in both COAD and READ in the Cancer Genome Atlas (TCGA) data analyzed by GEPIA^60^ (T: tumor, N: normal). Transcripts Per Million (TPM) counts per length of transcript (kb) per million reads mapped in RNA-seq.

NEU4 was down-regulated in all EMT-induced cancer stem-like cells colon cancer cell lines DLD1, HT29 and LS174T, but not NEU1, NEU2 and NEU3 (Fig. 1A). Because ~80% of colon cancer cell lines have some defects in the TGF-β signaling pathway through multiple mechanisms such as mutations in receptors, mutations in SMAD proteins, or overexpression of inhibitory SMAD6 or SMAD7 proteins^35^. LS174T or DLD1 cells have no response in TGF-β treatment^36,37^. We performed the TGF-β treatment with HT29 cells and found that NEU4 was also repressed in TGF-β mediated EMT (Fig. 1B). NEU1 and NEU2 are not able to remove sialic acid residues from sialyl Lewis X and sialyl Lewis A glycans^8^. NEU3 is a degradation enzyme for sialyl Lewis X but not for sialyl Lewis A^8^, but NEU3 is expressed at much lower levels relative to NEU4 in all colon cancer cells (Fig. 1C). According to the TCGA data from the GEPIA web site, NEU4 is down-regulated in tumors compared to normal tissues in both colon (COAD) and rectal (READ) cancers (Fig. 1D). Besides NEU2, which shows much lower expression in normal colon, COAD or READ, both NEU1 and NEU3 are up-regulated in COAD and READ. The database results are generally in line with our EMT data indicating that EGF and bFGF induced NEU1, NEU2, NEU3 but repressed NEU4.

TAp73 expression was much higher than ΔNp73 and ΔN’p73 in DLD1 and HT29 cells (Fig. S1A). In contrast, AP2α showed higher expression in HT29 cells than in DLD1 cells, but AP2γ expression was higher in DLD1 cells than in HT29 cells (Fig. S1B). NEU4 has long (NEU4L: NP_542779.2 and NP_001161071.1) and short (NEU4S: NP_001161074.1) forms, and NEU4S, but not NEU4L, is expressed in the colon^38^. Based on the NCBI reference sequences, NEU4L has two transcripts (NEU4 V1 and V2) and NEU4S has three transcripts (NEU4 V3, V4 and V5). NEU4 V3 is the dominant form of NEU4S in both the HT29 and DLD1 cell lines (Fig. S1C). NEU4 V3 and V4 transcripts use the same transcription start site and share the same promoter.

### p73 and AP2 regulate NEU4 to down-regulate sialyl Lewis X expression

**Figure 2:**
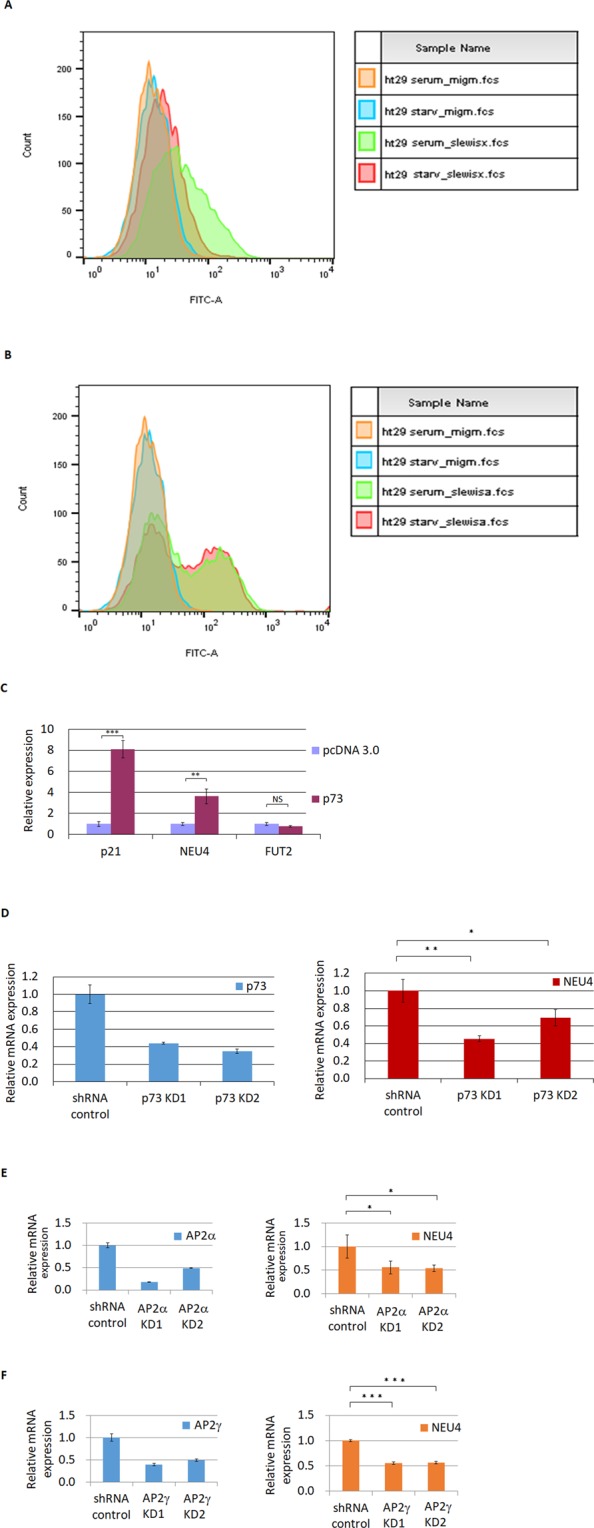
p73 and AP2 affect NEU4 expression. (**A**) sialyl Lewis X was down-regulated under starvation conditions in HT29 cells (orange line, isotype control under full serum culture; blue line, isotype control under starvation; green line, sialyl Lewis X under full serum culture; red line, sialyl Lewis X under starvation). (**B**) Sialyl Lewis A levels did not change under starvation conditions in HT29 cells (orange line, isotype control under full serum culture; blue line, isotype control under starvation; green line, sialyl Lewis A under full serum culture; red line, sialyl Lewis A under starvation). (**C**) NEU4, but not FUT2, mRNA was increased by p73 overexpression in HT29 cells. (**D,E**,**F**) Knock-down of p73 (**D**) or AP2α (**E**) in HT29 cells or of AP2γ in DLD1 cells (**F**) reduced NEU4 mRNA. (**C–F**) Results are displayed as mean ± SD, n = 3 (***p < 0.001, **p < 0.01, *p < 0.05).

Serum starvation in several p53-mutated colon cancer cell lines (HT29, DLD1, SW480 and SW837) activates p73 and overcomes dominant-negative functions of p53 to induce PUMA to induce cell apoptosis^39^. We performed serum starvation on the p53-mutant colon cancer cell line HT29 and found that the cancer-associated glycan sialyl Lewis X, but not sialyl Lewis A, was repressed under serum starvation relative to normal serum conditions (Fig. 2A,B). We over-expressed p73 in HT29 cells and found that only NEU4 mRNA, but not FUT2 mRNA, was increased by p73 (Fig. 2C), and knock-down of p73, AP2α or AP2γ reduced NEU4 expression (Fig. 2D–F). Over-expression of p53 activated p21, but not NEU4, in both the HT29 and LS174T cell lines, whereas p73 activated both p21 and NEU4 (Figs S2 and S3).

**Figure 3:**
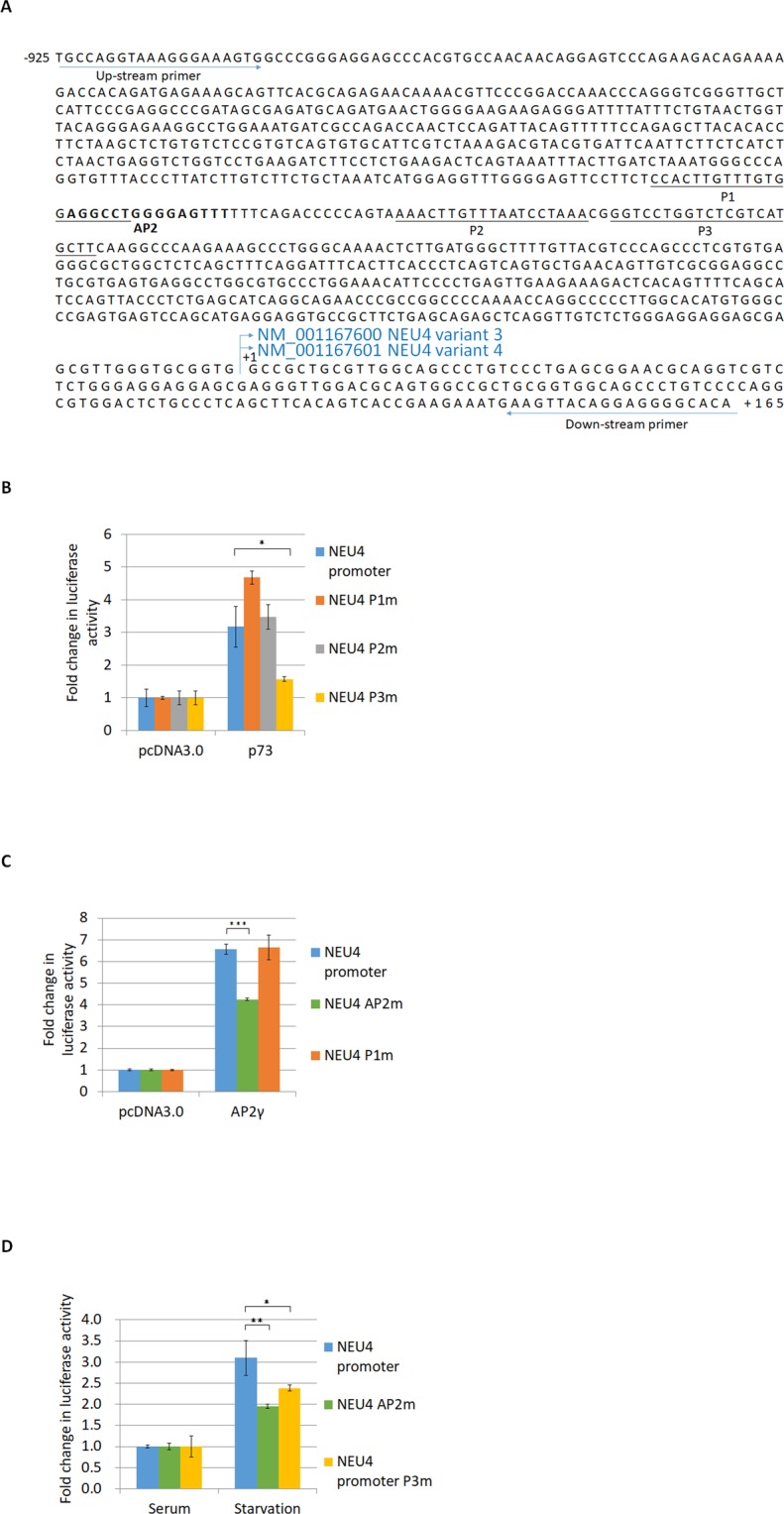
The NEU4 promoter is activated by p73 and AP2γ in HT29 cells. (**A**) Promoter sequence of NEU4 V34. P1–P3 are the potential binding sites for the p53 family members predicted by JASPAR software. The boldface letters represent the AP2 potential binding site predicted by JASPAR software. (**B**) Over-expression of p73 activated the NEU4 promoter, and mutation of P3 reduced the activation function of p73. (**C**) Over-expression of AP2γ activated the NEU4 promoter, and mutation of the AP2 potential binding site, but not mutation of P1, reduced the activation function of AP2γ. (**D**) Starvation conditions activated the NEU4 promoter, and this effect was partially reduced when the promoter contained P3 and AP2 mutations. (**B**–**D**) Results are displayed as mean ± SD, n = 3 (***p < 0.001, **p < 0.01, *p < 0.05).

**Figure 4:**
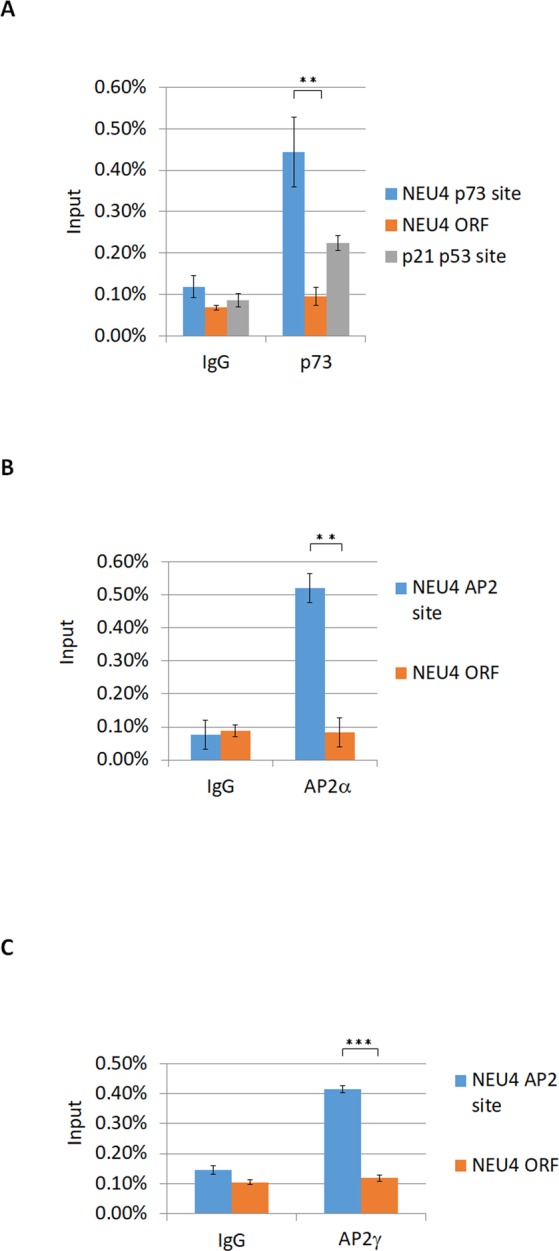
p73 and AP2 bind to the NEU4 promoter in ChIP assays. (**A**) p73 bound the NEU4 and p21 promoters, but not the open reading frame of NEU4, in HT29 cells. (**B**) AP2α bound the NEU4 promoter, but not the open reading frame of NEU4, in HT29 cells. (**C**) AP2γ bound the NEU4 promoter, but not the open reading frame of NEU4, in DLD1 cells. (**A**–**C**) Results are displayed as mean ± SD, n = 3 (***p < 0.001, **p < 0.01).

**Figure 5:**
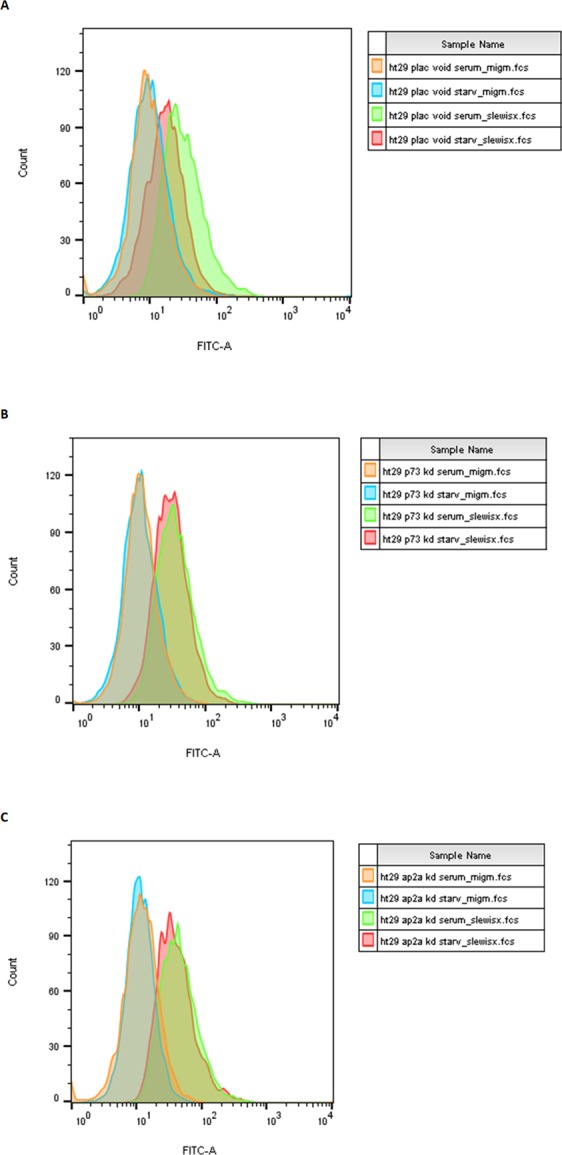
p73 or AP2α knock-down rescues starvation-mediated sialyl Lewis X repression. (**A**) Starvation repressed sialyl Lewis X expression in HT29 cells infected with a scrambled shRNA (orange line, isotype control with cells infected with a control scrambled shRNA cultured with full serum; blue line, isotype control with cells infected with a control scrambled shRNA under starvation; green line, sialyl Lewis X staining with cells infected with a control scrambled shRNA cultured with full serum; red line, sialyl Lewis X staining with cells infected with a control scrambled shRNA cultured under starvation). (**B**) p73 knock down (p73 KD1 cells, as in Fig. 2D) rescued the starvation-mediated sialyl Lewis X repression (orange line, isotype control with p73 KD1 cells cultured with full serum; blue line, isotype control with p73 KD1 cells cultured under starvation; green line, sialyl Lewis X staining with p73 KD1 cells cultured with full serum; red line, sialyl Lewis X staining with p73 KD1 cells cultured under starvation). (**C**) AP2α knock down (AP2α KD1 cells, as in Fig. 2E) rescued starvation-mediated sialyl Lewis X repression (orange line, isotype control for AP2α KD1 cells cultured with full serum; blue line, isotype control for AP2α KD1 cells cultured under starvation; green line, sialyl Lewis X staining for AP2α KD1 cells cultured with full serum; red line, sialyl Lewis X staining for AP2α KD1 cells cultured under starvation).

We cloned the NEU4 V34 promoter of 1090 bp (−925~+165) into pGL3 basic to carry out a reporter assay and found that serum starvation activated the NEU4 promoter. According to Jaspar database prediction^40^, there are three putative p73 binding sites and one AP2 binding site in the NEU4 promoter (Fig. 3A). Mutation of one of the p73 sites and the only AP2 binding site reduced p73- and AP2γ-induced enhancement of NEU4 promoter reporter activity, respectively (Fig. 3B,C). Up-regulation of the NEU4 promoter mediated by serum starvation was diminished by mutation of a p73 or AP2 binding site (Fig. 3D). Binding of p73, AP2α or AP2γ to the NEU4 promoter was confirmed by chromatin immunoprecipitation (ChIP) assays (Fig. 4A–C). We performed the sialidases activity assays in HT29 and LS174T cells, and results showed that p73 could enhance the sialidase activity compared to vector only or p53 (Fig. S4). Over-expression of a Flag-tagged NEU4 construct down-regulated sialyl Lewis X expression in HT29 cells (Fig. S5A and S5B). We used a colon cancer cell line that has wild-type p53, LS174T, and found that over-expression of p73, but not of p53, down-regulated sialyl Lewis X expression (Fig. S6A). In order to prove p73 directly represses sialyl Lewis X through NEU4, we introduced NEU4 siRNA in p73 over-expressing cells and found that repressive effect of p73 for sialyl Lewis X expression were withdrawn in NEU4 knocked-down cells (Fig. S6B). The p73 mediated NEU4 expression was also decreased in these NEU4 knocked-down cells (Fig. S6C). Serum starvation in HT29 cells in which p73 or AP2 had been knocked down did not result in notable repression of sialyl Lewis X levels (Fig. 5). These results indicate that p73 and AP2 are able to regulate NEU4 and influence starvation-mediated sialyl Lewis X expression.

### p73 interacts with AP2 through a different key residue relative to p53 and p63

**Figure 6:**
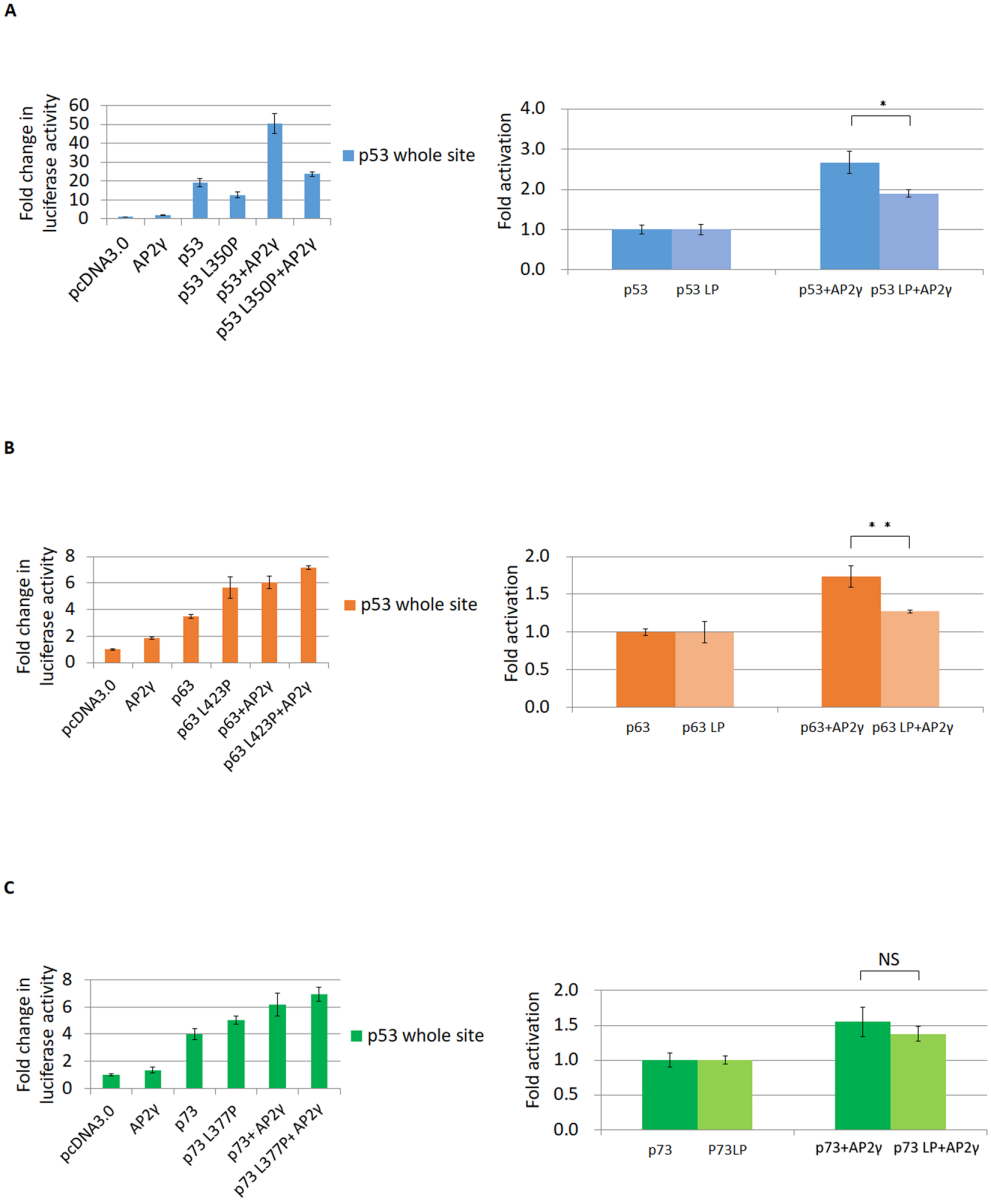
Synergistic effect of AP2 and p53 family members on the activation of the p53 responsive element. (**A**) Co-expression of p53 and AP2γ up-regulated expression of the reporter with p53 whole site to a greater extent than p53 alone, but co-expression of p53 with the L350P mutation (p53LP) and AP2γ only slightly up-regulated expression of the p53 whole site reporter relative to expression of p53LP alone in H1299 cells. (**B**) Co-expression of p63 and AP2γ increased expression of the p53 whole site reporter much higher than p63 alone, but co-expression of p63LP and AP2γ led to only a slight increase of p53 whole site reporter expression relative to p63LP alone. (**C**) Co-expression of p73 and AP2γ increased expression of the p53 whole site reporter higher than p73 alone, and co-expression p73LP and AP2γ also up-regulated expression of the p53 whole site reporter higher than p73LP alone. (**A**–**C**) Results are displayed as mean ± SD, n = 3 (**p < 0.01, *p < 0.05). (NS, statistically not significant.

**Figure 7:**
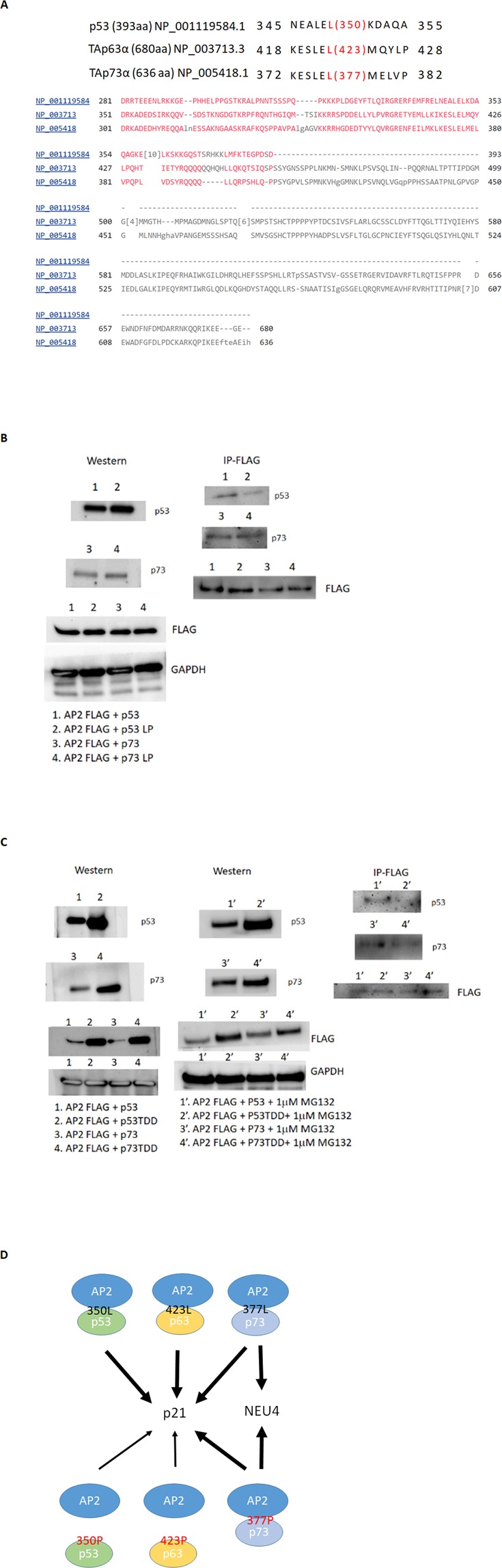
p73 exhibits a protein-protein interaction with AP2. (**A**) Partial sequence alignment within the tetramerization domains of p53 family members. COBALT^61^ was used for multiple protein sequences alignment of the C-terminal of p53 family members. (**B**) Flag-tagged AP2γ pulled down p53 and p73, albeit at lower amounts when p53LP was used (but not p73LP) in H1299 cells. (**C**) p53TDD and p73TDD (tetramerization domain deletion) influenced Flag-AP2γ expression, and this effect was reduced by adding MG132 (1 μM). Under treatment of MG132, p53TDD and p73TDD interaction with AP2γ were much less than that with wild-type p53 and p73. (**D**) A schematic figure explaining how the p53 WT or LP mutation regulates the p21 and NEU4 which contain both AP2 and p53 responsive elements. p53 family members can interact with AP2 to regulate down-stream genes. LP mutations of p53 and p63 lose the interaction with AP2 to regulate p21, but p73 LP mutant still interacts with AP2 to facilitate p21 and NEU4.

p53 interacts with AP2 to enhance p21 promoter activity^18^, and the p53 L350P mutation and tetramerization domain deletion (TDD) disrupt this interaction with AP2^41^. p53 and AP2γ, acting synergistically, activated a p53 consensus sequence in p53 null H1299 cells, and this effect was reduced by the L350P mutation (Fig. 6A). p53, p63 and p73 all have a leucine residue—p53 L350, TAp63α L423 and TAp73α L377, respectively—in their tetramerization domain^42^. In addition, TAp63α L423P, but not TAp73α L377P, disrupted the synergistic effect with AP2γ (Fig. 6B,C). Interaction of TAp73α and AP2γ was reduced in cells expressing TAp73α TDD but not in those expressing TAp73α L377P (Fig. 7B,C). This means that L377 of p73 is not the key residue that interacts with AP2, unlike L350 of p53 and L423 of p63. Co-expression of TAp73α and AP2γ increased expression of NEU4 synergistically relative to their individual expression, with a greater effect in LS174T cells as compared with HT29 cells (Fig. S7A,B). Therefore, we propose that p73 and AP2γ synergistically activate the NEU4 promoter.

## Discussion

NEU4 can reportedly regulate several cancer-associated glycans, such as sialyl Lewis A, sialyl Lewis X, and PSA-NCAM^8,9^, all three of which are highly expressed in colon cancers^2–5^. Sialyl Lewis X, as well as sialyl Lewis A, serves as a ligand for vascular selectins, the cell adhesion molecules expressed on endothelial cells. Sialyl Lewis X mediates adhesion of cancer cells to vascular endothelial cells and facilitates hematogenous metastasis^33,34^. Polysialylated NCAM is associated with aggressiveness and poor clinical outcome in cancers^43^. In contrast, both p73 and AP2 are tumor suppressors, but their roles in glycan expression remain unknown. Here we found that serum starvation in colon cancer cells reduced the amount of sialyl Lewis X but not sialyl Lewis A. NEU4 may regulate both sialyl Lewis A and sialyl Lewis X, but starvation-related NEU4-mediated up-regulation did not influence sialyl Lewis A. This means that serum starvation may influence other key glycan-synthesizing enzymes that are specific for sialyl Lewis A, such as ST3GAL3, B3GALT5 or FUT3^44–46^, to overcome the NEU4 effect on sialyl Lewis A expression. Starvation up-regulated expression of B3GALT5 and FUT3, but not ST3GAL3 (Fig. S8).

This study proved that p73 and AP2 regulate NEU4 and influence starvation-mediated sialyl Lewis X expression. Because p53 shows a higher frequency of mutation in colon cancer as compared with other cancers, activation of p73 to replace the loss of p53 function is a good strategy for anti-cancer therapy. There are several p73 activators that could be used in p53-mutated cells^47,48^. To use these p73 activators to activate NEU4 to repress cancer-associated glycans may be a good strategy for developing colon cancer therapies.

Here we found that p73 interacts with AP2 through its tetramerization domain, as does p53 and p63, but the key residue within the tetramerization domain is different (Fig. 7A,B). Both p53 L350P and TAp63α L423 blocked the interaction with AP2, but the TAp73α L377P could not (Fig. 7B). This makes the down-stream genes of the p73 and AP2 synergistic activation difficult to study. Co-expression of p53 (or p63) and AP2 should activate the down-stream genes more effectively than p53 L350P (or TAp63α L423P) and AP2, but this strategy could not be used for p73. Deletion of the tetramerization domain rather than using the LP mutation to study the activation function is not feasible, because all the p53 family members lose their activation function if they cannot form a tetramer^49,50^. A reporter gene in combination with the p53 consensus sequence is a possible method for studying the synergistic effect of AP2 and p53 to activate genes^41^ (Fig. 6). Co-expression of these transcription factors should activate the gene synergistically, relative to the activation resulting from individual transcription factors alone^51,52^. We found that synergistic up-regulation of NEU4 by p73 and AP2 is higher in LS174T (wild-type p53) than HT29 (mutated p53) cells. Therefore, cells with mutated p53 may block the synergistic effect of AP2 and p73 to active NEU4.

NEU4 has both long (NEU4L) and short (NEU4S) forms, and only NEU4S but not NEU4L is expressed in colon cells^38^. Here we showed that p73 activates the NEU4 short form in colon cancer cells. In neural cells, NEU4L and NEU4S are almost equally expressed^38^. Endogenous TAp73 levels increase in differentiated neuroblastomas after retinoic acid treatment, and exogenous TAp73 isoforms can induce neuronal differentiation^53^. One of the NEU4 target glycoproteins, PSA-NCAM, has an important role in neural cell migration^54^, differentiation^55^, neurogenesis^54^ and the developing thalamus^56^. Therefore, the role that p73 has with respect to NEU4S (or NEU4L) expression may also have an effect on neural development, which needs further investigation.

## Materials and Methods

### Cell lines and cell culture

Human colon cancer cell lines DLD1, HT29 and LS174T and the non-small cell lung carcinoma cell line H1299 were maintained at 37 °C with 5% CO_2_ in RPMI 1640 or DMEM (Invitrogen, Carlsbad, CA, USA) supplemented with 10% FBS (Invitrogen), 100 U/ml penicillin and 100 μg/ml streptomycin (both from Invitrogen). For treatment with EGF and bFGF, recombinant human EGF (R&D; 20 ng/ml) and bFGF (R&D; 10 ng/ml) were added to serum-free DMEM/F12 (Invitrogen) with 0.4% bovine serum albumin (Sigma), N-2 MAX Media Supplement (R&D), B27 Supplement (Invitrogen), 100 U/ml penicillin and 100 μg/ml streptomycin (Invitrogen) as described^4,57^. For treatment with TGF-β, cells were cultured in medium without serum for 1 day, then treated with recombinant human TGF-β1 (HEK293 derived, Peprotech, 10 ng/ml) in serum-free medium for 2 days as described^58^. For cell starvation, cells were serum starved in serum-free DMEM with 100 U/ml penicillin and 100 μg/ml streptomycin for 3 days.

### Flow cytometry

The cultured cells were harvested (5 × 10^5^ cells in each tube), centrifuged at 300 *g* for 5 min and incubated with an antibody against sialyl Lewis X (clone SNH-3, murine IgM), as described previously^3^ or against sialyl Lewis A (clone 2D3, murine IgM)^2^ at 4 °C for 30 min. Then the cells were washed with 1 ml FACS buffer (PBS containing 2% FCS) and stained with FITC-conjugated secondary antibody (Biolegend, San Diego, CA, USA, RMM-1) at 4 °C for 20 min. The cells were washed with 1 ml FACS buffer two times, resuspended in 0.4 ml FACS buffer and kept in the dark on ice until FACS analysis. The cells were passed through a mesh and analyzed with a FACSCalibur (BD Biosciences, San Jose, CA, USA).

### Reporter constructs and luciferase assays

The NEU4V34 promoter of 1090 bp (−925~+165) (Homo sapiens chromosome 2, GRCh38.p12 Primary Assembly. NC_000002: 241808140.0.241809229) was amplified from colon cancer cell genomic DNA using forward primer 5′-TGCCAGGTAAAGGGAAAGTG-3′ and reverse primer 5′-TGTGCCCCTCCTGTAACTTC-3′ and was cloned to up-stream of the firefly luciferase reporter gene in pGL3 basic (Promega). We co-transfected pGL3-NEU4 firefly luciferase plasmid or p53 whole site in the pGL3 promoter vector^49^ and pRL-SV40 Renilla luciferase plasmids (Promega) into cells. Cells were harvested at 24 h post-transfection in 0.25 ml of reporter lysis buffer and were assayed for gene expression with the Dual-Luciferase Reporter Assay System (Promega). Firefly luciferase activity was normalized to Renilla luciferase activity, and the data are presented as the mean ± standard deviation from three independent experiments, each of which was performed in triplicate.

### Co-immunoprecipitation

Cells were scraped into NP40 lysis buffer containing protease and phosphatase inhibitor cocktails (Merck). After cellular debris was removed by centrifugation, the pre-cleared extract was incubated with anti-FLAG M2 Magnetic Beads (Sigma) for 2–3 h at 4 °C using constant rotation. The beads were collected in magnetic separation rack and were washed three times in wash buffer (150 mM NaCl; 50 mM Tris, pH 7.5; 1 mM EDTA; 0.5% (w/v) NP40; 10% glycerol). Bound proteins were eluted with 20 μl of 500 μg/ml Flag peptide (Sigma) in elution buffer (150 mM NaCl; 50 mM Tris, pH 7.5; 1 mM EDTA; 0.05% NP40; 10% glycerol) for 10 min at 4 °C and were detected by using anti-p53 (sc-126, Santa Cruz) or anti-p73 (ab14430, Abcam) by western blot analysis.

### ChIP assay

We used EZ-Magna ChIP A/G Chromatin Immunoprecipitation Kit (Millipore) to perform the ChIP assays. We used ChIP-grade anti-p73 (ab14430) along with anti-AP2α and -AP2γ (sc-184X and sc-8977x, respectively; Santa Cruz). The fixed DNA was sheared with a Bioruptor Pico (Diagenode), and precipitated DNA was quantified with a Bio-Rad real-time thermal cycler CFX96.

### shRNA infection

We purchased shRNA clones of pLKO.1-shRNA-AP2α (AP2α KD1, TRCN0000004924; AP2α KD2, TRCN0000004926), pLKO.1-shRNA-AP2γ (AP2γ KD1, TRCN0000019756; AP2γ KD2, TRCN0000019748) and pLKO.1-shRNA-p73 (p73 KD1, TRCN0000006507; p73 KD2, TRCN0000006508) from the National RNAi Core Facility, Academia Sinica. All of these vectors were purified and sent to the National RNAi Core Facility to package the virus. Virus packaged with the pLKO scrambled shRNA (pLAS.Void) was purchased from the National RNAi Core Facility. All virus-packaged shRNAs were infected into HT29 or DLD1 cells.

### Real-Time Quantitative PCR (qPCR) and primers

Total cellular RNA was extracted with TRIzol reagent (Invitrogen). First-strand cDNA for mRNAs was prepared from 1 μg total RNA with the Maxima H Minus FirstStrand cDNA Synthesis kit (Thermo Scientific, Waltham, MA, USA). For conventional qPCR, cDNA samples or DNA samples from ChIP assays were mixed with EvaGreen Supermix (Bio-Rad, Hercules, CA, USA) and primers (Tables S1 and S2). The amplification for all qPCRs was monitored using the CFX Connect Real-Time PCR System (Bio-Rad). Relative transcript levels were calculated as 2^−ΔΔ^CT

### Site-directed mutagenesis

The NEU4 P1m, NEU4 P2m, NEU4 P3m, NEU4 AP2m, p53 L350P, TAp63 alpha L423P, TAp73 alpha L377, p53TDD and p73TDD point mutations or deletion clones were created by site-directed mutagenesis (Phusion Site-Directed Mutagenesis Kit, Finnzymes). The following primers were used: NEU4 P1m, 5′-AAAGAACTGGAGAAGGAACTCCCCAAA-3′ and 5′-GTGGAGCCCTGGGGAGTTTTTTC-3′; NEU4 P2m, 5′-GGTCTGAAAAAACTCCCCAGG-3′ and 5′-CCCAGTAAAAGTTCTTTAATGCTAAACGGGTC-3′; NEU4 P3m, 5′-AGAGCACGACCCGTTTAGGATTAAGCA-3′ and 5′-CGTGATCCTTCAAGGCCCAAGAAA-3′; NEU4 AP2m, 5′-CATTTCTCCACAAACAAGTGGAGAAGGA-3′ and 5′-GGAAAGTTTTTTCAGACCCCCAGTAAAAC-3′; p53 L350P, 5′-GGGCATCCTTGGGTTCCAAGGC-3′ and 5′-AGGCTGGGAAGGAGCCAGGG-3′; TAp63 alpha L423P, 5′-GAAGGTACTGCATGGGTTCCAGGGACTC-3′ and 5′-CTCAGCACACAATTGAAACGTACAGGC-3′; TAp73 alpha L377, 5′-TGCCGCAGCCACTGGTGGA-3′ and 5′-CCAACTCCATCGGCTCCAGGCTC-3′; p53TDD, 5′-CAGTGGTTTCTTCTTTGGCTGGG-3′ and 5′-GGGGGGAGCAGGGCTCACT-3′; p73TDD, 5′-TCCATGCCGCCGCTTCTTC-3′ and 5′-GACTCCTATCGGCAGCAGCAGC-3′. All constructs were checked by DNA sequencing.

### Sialidases activity assay

NEU4 sialidase activity was analyzed with 2′-(4-methylumbelliferyl)-α-D-N-acetylneuraminic acid (4MU-NeuAc; Sigma) as a substrate^59^. Reactions were set up in triplicate using 30 μg of total proteins with 50 mM Na citrate/phosphate buffer (pH 3.2), 0.1 mM 4MU-NeuAc, and 6 mg/mL BSA in a final volume of 100 μl, and incubated at 37 °C for 30 min. Reactions were stopped by addition of 1 ml of 0.2 M glycine/NaOH (pH 10.8). The fluorescence associated with the release of 4-MU was measured at the excitation wavelength of 365 nm and an emission wavelength of 445 nm with an ARVO MX plate reader (Perkin–Elmer). The sialidase from Arthrobacter ureafaciens (Roche) was used in the assay to obtain a standard curve.

### siRNA transfection

Cultured cells (1 × 10^6^) were added to 90 µl BTXpress electroporation buffer (BTX, Holliston, MA) with 3 µL (10 µM) of control siRNA (sc-37007; Santa Cruz) or NEU4 siRNA (sc-94619; Santa Cruz) and 3 µg of pcDNA3.0 or p73 vectors. The mixture was transferred into a 2-mm BTX Gap Cuvette and electroporated (80 V, 13 ms, two pulses, 1-s interval) using a BTX Gemini X2 Electroporation system (BTX, Holliston, MA). The cells were harvested 48 h after siRNA transfection to evaluate NEU4 mRNA or sialyl Lewis X expression.

### Statistical analysis

Potential statistical differences between two groups were assessed with the Student’s *t* test (two tailed). All results are presented as the mean ± SD. P value of less than 0.05 was considered statistically significant (*P < 0.05, **P < 0.01, ***P < 0.001).

## Supplementary information

Supplementary Material

## References

[1] Li BQ (2013). An ensemble prognostic model for colorectal cancer. PLoS One.

[2] Miyazaki K (2004). Loss of disialyl Lewis(a), the ligand for lymphocyte inhibitory receptor sialic acid-binding immunoglobulin-like lectin-7 (Siglec-7) associated with increased sialyl Lewis(a) expression on human colon cancers. Cancer Res.

[3] Yusa A, Miyazaki K, Kimura N, Izawa M, Kannagi R (2010). Epigenetic silencing of the sulfate transporter gene DTDST induces sialyl Lewisx expression and accelerates proliferation of colon cancer cells. Cancer Res.

[4] Sakuma K, Aoki M, Kannagi R (2012). Transcription factors c-Myc and CDX2 mediate E-selectin ligand expression in colon cancer cells undergoing EGF/bFGF-induced epithelial-mesenchymal transition. Proc Natl Acad Sci USA.

[5] Fernández-Briera A, García-Parceiro I, Cuevas E, Gil-Martín E (2010). Effect of human colorectal carcinogenesis on the neural cell adhesion molecule expression and polysialylation. Oncology.

[6] Ye J (2012). Enrichment of colorectal cancer stem cells through epithelial-mesenchymal transition via CDH1 knockdown. Mol Med Rep.

[7] Fan F (2012). Overexpression of snail induces epithelial-mesenchymal transition and a cancer stem cell-like phenotype in human colorectal cancer cells. Cancer Med.

[8] Shiozaki K, Yamaguchi K, Takahashi K, Moriya S, Miyagi T (2011). Regulation of sialyl Lewis antigen expression in colon cancer cells by sialidase NEU4. J Biol Chem.

[9] Takahashi K (2012). Sialidase NEU4 hydrolyzes polysialic acids of neural cell adhesion molecules and negatively regulates neurite formation by hippocampal neurons. J Biol Chem.

[10] Yamanami H (2007). Down-regulation of sialidase NEU4 may contribute to invasive properties of human colon cancers. Cancer Sci.

[11] Wilde A (1999). EGF receptor signaling stimulates SRC kinase phosphorylation of clathrin, influencing clathrin redistribution and EGF uptake. Cell.

[12] Aqeilan RI (2004). Functional association between Wwox tumor suppressor protein and p73, a p53 homolog. Proc Natl Acad Sci USA.

[13] Aqeilan RI (2004). Physical and functional interactions between the Wwox tumor suppressor protein and the AP-2gamma transcription factor. Cancer Res.

[14] Li H, Goswami PC, Domann FE (2006). AP-2gamma induces p21 expression, arrests cell cycle, and inhibits the tumor growth of human carcinoma cells. Neoplasia.

[15] Kerschgens J (2011). Protein-binding microarray analysis of tumor suppressor AP2α target gene specificity. PLoS One.

[16] Collavin L, Lunardi A, Del Sal G (2010). p53-family proteins and their regulators: hubs and spokes in tumor suppression. Cell Death Differ.

[17] Ropponen KM (2001). Expression of transcription factor AP-2 in colorectal adenomas and adenocarcinomas; comparison of immunohistochemistry and *in situ* hybridisation. J Clin Pathol.

[18] McPherson LA, Loktev AV, Weigel RJ (2002). Tumor suppressor activity of AP2alpha mediated through a direct interaction with p53. J Biol Chem.

[19] McDade SS (2012). Genome-wide analysis of p63 binding sites identifies AP-2 factors as co-regulators of epidermal differentiation. Nucleic Acids Res.

[20] El-Deiry WS (1993). WAF1, a potential mediator of p53 tumor suppression. Cell.

[21] Hermeking H (1997). 14-3-3 sigma is a p53-regulated inhibitor of G2/M progression. Mol Cell.

[22] Nigro JM (1989). Mutations in the p53 gene occur in diverse human tumour types. Nature.

[23] Iacopetta B (2003). TP53 mutation in colorectal cancer. Hum Mutat.

[24] Blagosklonny MV (1997). Loss of function and p53 protein stabilization. Oncogene.

[25] Levrero M (2000). The p53/p63/p73 family of transcription factors: overlapping and distinct functions. J Cell Sci.

[26] Pützer BM, Tuve S, Tannapfel A, Stiewe T (2003). Increased DeltaN-p73 expression in tumors by upregulation of the E2F1-regulated, TA-promoter-derived DeltaN’-p73 transcript. Cell Death Differ.

[27] Koster MI, Kim S, Mills AA, DeMayo FJ, Roop DR (2004). p63 is the molecular switch for initiation of an epithelial stratification program. Genes Dev.

[28] Su XL, Ouyang XH, Yan MR, Liu GR (2009). p73 expression and its clinical significance in colorectal cancer. Colorectal Dis.

[29] Herreros-Villanueva M, Muñiz P, García-Girón C, Cavia-Saiz M, Del Corral M (2010). J. TAp73 is one of the genes responsible for the lack of response to chemotherapy depending on B-Raf mutational status. J Transl Med.

[30] Díaz R (2010). Differential regulation of TP73 isoforms by 1α,25-dihydroxyvitamin D3 and survivin in human colon and breast carcinomas. Genes Chromosomes Cancer.

[31] Wang W, Kim SH, El-Deiry WS (2006). Small-molecule modulators of p53 family signaling and antitumor effects in p53-deficient human colon tumor xenografts. Proc Natl Acad Sci USA.

[32] Chan WM, Siu WY, Lau A, Poon RY (2004). How many mutant p53 molecules are needed to inactivate a tetramer?. Mol Cell Biol.

[33] Kannagi R (2010). Altered expression of glycan genes in cancers induced by epigenetic silencing and tumor hypoxia: clues in the ongoing search for new tumor markers. Cancer Sci.

[34] Pinho SS, Reis CA (2015). Glycosylation in cancer: mechanisms and clinical implications. Nat Rev Cancer.

[35] Jung B, Staudacher JJ, Beauchamp D (2017). Transforming Growth Factor β Superfamily Signaling in Development of Colorectal Cancer. Gastroenterology.

[36] Pino MS (2010). Epithelial to mesenchymal transition is impaired in colon cancer cells with microsatellite instability. Gastroenterology.

[37] Hirakawa M (2014). Fucosylated TGF-β receptors transduces a signal for epithelial-mesenchymal transition in colorectal cancer cells. Br J Cancer.

[38] Yamaguchi K (2005). Evidence for mitochondrial localization of a novel human sialidase (NEU4). Biochem J.

[39] Ming L (2008). Sp1 and p73 activate PUMA following serum starvation. Carcinogenesis.

[40] Mathelier A (2014). JASPAR 2014: an extensively expanded and updated open-access database of transcription factor binding profiles. Nucleic Acids Res.

[41] Stabach PR, Thiyagarajan MM, Woodfield GW, Weigel RJ (2006). AP2alpha alters the transcriptional activity and stability of p53. Oncogene.

[42] Chène P (2001). The role of tetramerization in p53 function. Oncogene.

[43] Falconer RA, Errington RJ, Shnyder SD, Smith PJ, Patterson LH (2012). Polysialyltransferase: a new target in metastatic cancer. Curr Cancer Drug Targets.

[44] Aronica A (2017). Unexpected distribution of CA19.9 and other type 1 chain Lewis antigens in normal and cancer tissues of colon and pancreas: Importance of the detection method and role of glycosyltransferase regulation. Biochim Biophys Acta.

[45] Trinchera, M., Aronica, A. & Dall’Olio, F. Selectin Ligands Sialyl-Lewis a and Sialyl-Lewis x in Gastrointestinal Cancers. *Biology (Basel)* 6, 10.3390/biology6010016 (2017). 10.3390/biology6010016 PMC5372009 28241499

[46] Orntoft TF (1996). Influence of Lewis alpha1-3/4-L-fucosyltransferase (FUT3) gene mutations on enzyme activity, erythrocyte phenotyping, and circulating tumor marker sialyl-Lewis a levels. J Biol Chem.

[47] Kravchenko JE (2008). Small-molecule RETRA suppresses mutant p53-bearing cancer cells through a p73-dependent salvage pathway. Proc Natl Acad Sci USA.

[48] Zhang S (2015). Small-Molecule NSC59984 Restores p53 Pathway Signaling and Antitumor Effects against Colorectal Cancer via p73 Activation and Degradation of Mutant p53. Cancer Res.

[49] Cai BH (2009). Functional four-base A/T gap core sequence CATTAG of P53 response elements specifically bound tetrameric P53 differently than two-base A/T gap core sequence CATG bound both dimeric and tetrameric P53. Nucleic Acids Res.

[50] McLure KG, Lee PW (1998). How p53 binds DNA as a tetramer. EMBO J.

[51] Lee JM, Libermann TA, Cho JY (2010). The synergistic regulatory effect of Runx2 and MEF transcription factors on osteoblast differentiation markers. J Periodontal Implant Sci.

[52] Zhou W (2015). A novel TBX5 loss-of-function mutation associated with sporadic dilated cardiomyopathy. Int J Mol Med.

[53] De Laurenzi V (2000). Induction of neuronal differentiation by p73 in a neuroblastoma cell line. J Biol Chem.

[54] Bonfanti L (2006). PSA-NCAM in mammalian structural plasticity and neurogenesis. Prog Neurobiol.

[55] Vutskits L (2001). PSA-NCAM modulates BDNF-dependent survival and differentiation of cortical neurons. Eur J Neurosci.

[56] Mazzetti S, Ortino B, Inverardi F, Frassoni C, Amadeo A (2007). PSA-NCAM in the developing and mature thalamus. Brain Res Bull.

[57] Chao CC (2017). Downregulation of miR-199a/b-5p is associated with GCNT2 induction upon epithelial-mesenchymal transition in colon cancer. FEBS Lett.

[58] Ma M (2016). MiR-487a Promotes TGF-β1-induced EMT, the Migration and Invasion of Breast Cancer Cells by Directly Targeting MAGI2. Int J Biol Sci.

[59] Bigi A (2013). A proline-rich loop mediates specific functions of human sialidase NEU4 in SK-N-BE neuronal differentiation. Glycobiology.

[60] Tang Z, Li C, Kang B, Gao G, Zhang Z (2017). GEPIA: a web server for cancer and normal gene expression profiling and interactive analyses. Nucleic Acids Res.

[61] Papadopoulos JS, Agarwala R (2007). COBALT: constraint-based alignment tool for multiple protein sequences. Bioinformatics.

