# Supplementary material for: Synergistic activation of the NEU4 promoter by p73 and AP2 in colon cancer cells
Source: Sci Rep. 2019 Jan 30;9:950. doi: 10.1038/s41598-018-37521-7 (PMC6353964; doi:10.1038/s41598-018-37521-7)
Supplement: Supplementary file 1 — Supplementary Material [file 41598_2018_37521_MOESM1_ESM.docx]

**Supplemental Materials for**

**"Synergistic activation of the NEU4 promoter by p73 and AP2 in colon cancer cells"**

**Bi-He Cai, Po-Han Wu, Chi-Kan Chou, Hsiang-Chi Huang, Chia-Chun Chao, Hsiao-Yu Chung, Hsueh-Yi Lee, Jang-Yi Chen, Reiji Kannagi**

**
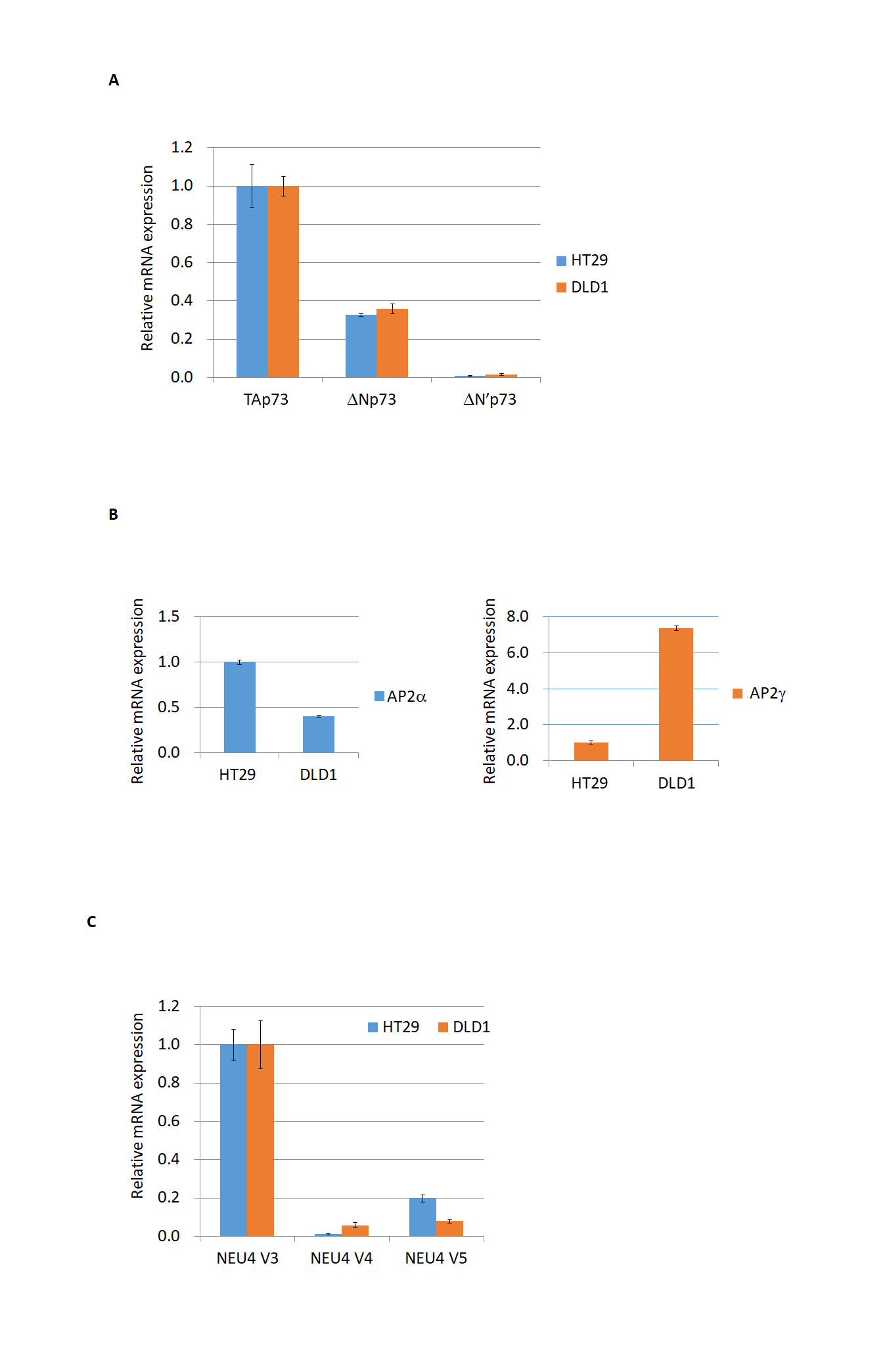
**

**Fig S1. Expression patterns of p73, AP2 and NEU4 in colon cancer cells.** (A) TAp73 showed the highest expression among the p73 isoforms in colon cancer cells. (B) AP2α showed much higher expression in HT29 cells than in DLD1 cells. AP2γ showed much higher expression in DLD1 cells than in HT29 cells. (C) NEU4V3 showed the highest expression as compared with V4 and V5 in colon cancer cells. (A-C) Results are displayed as mean ± SD, n=3


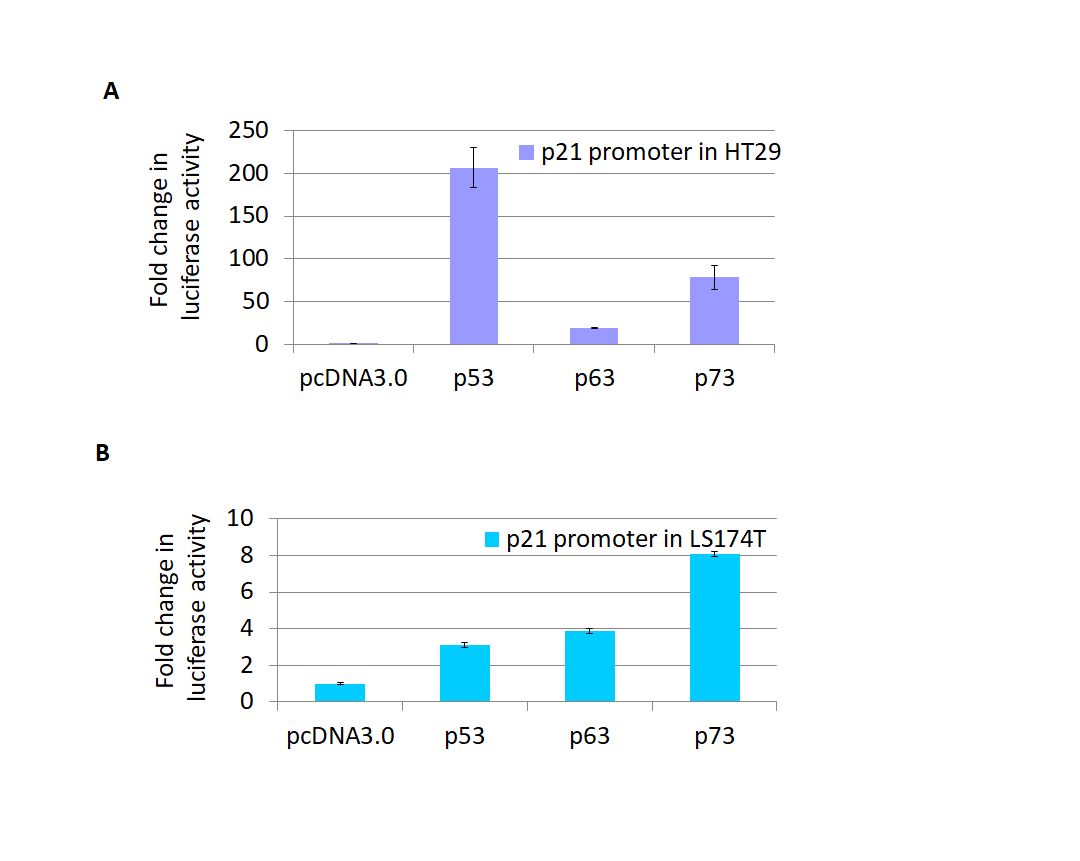


**Fig S2. p53 and p73 can activate p21 in colon cancer cells.** Results from luciferase reporter assays with over-expression of pcDNA3.0 (empty vector), p53, p63 and p73 are shown. (A, B) All p53 family members tested here activated the p21 promoter in HT29 cells (A) and in LS174T cells (B). Results are displayed as mean ± SD, n=3

**
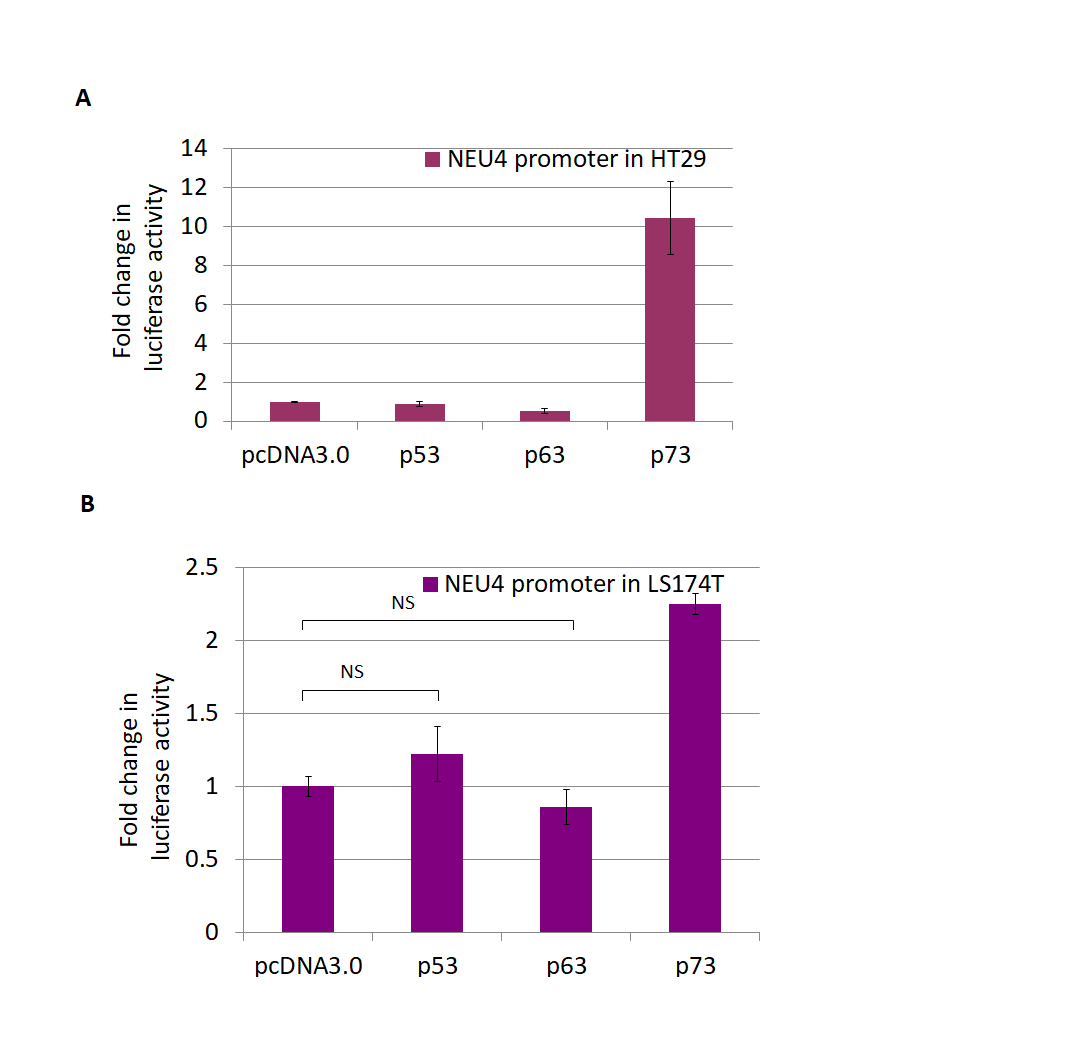
**

**Fig S3. p73, but not p53, can activate NEU4 in colon cancer cells.** (A, B) p73, but not p53 or p63, activated the NEU4 promoter in HT29 cells (A) and in LS174T cells (B). Results are displayed as mean ± SD, n=3 (NS, statistically not significant)

**
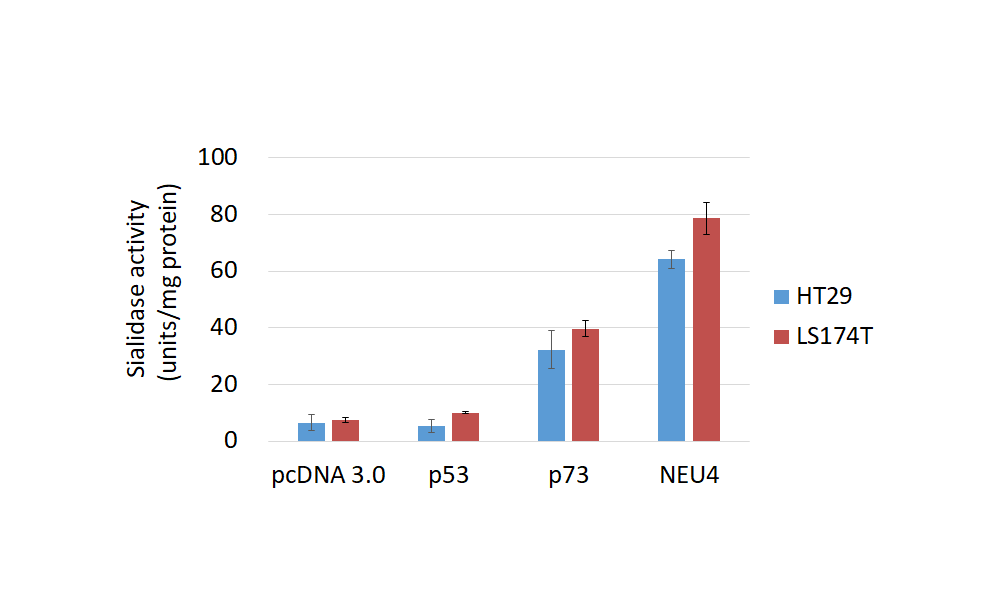
**

**Fig S4. p73, but not p53, can increase sialidase activity.** p73 and NEU4 (positive control) could up-regulate the sialidase activity compared to p53 or vector only (negative control) in HT29 and LS174T cells. Results are displayed as mean ± SD, n=3

**
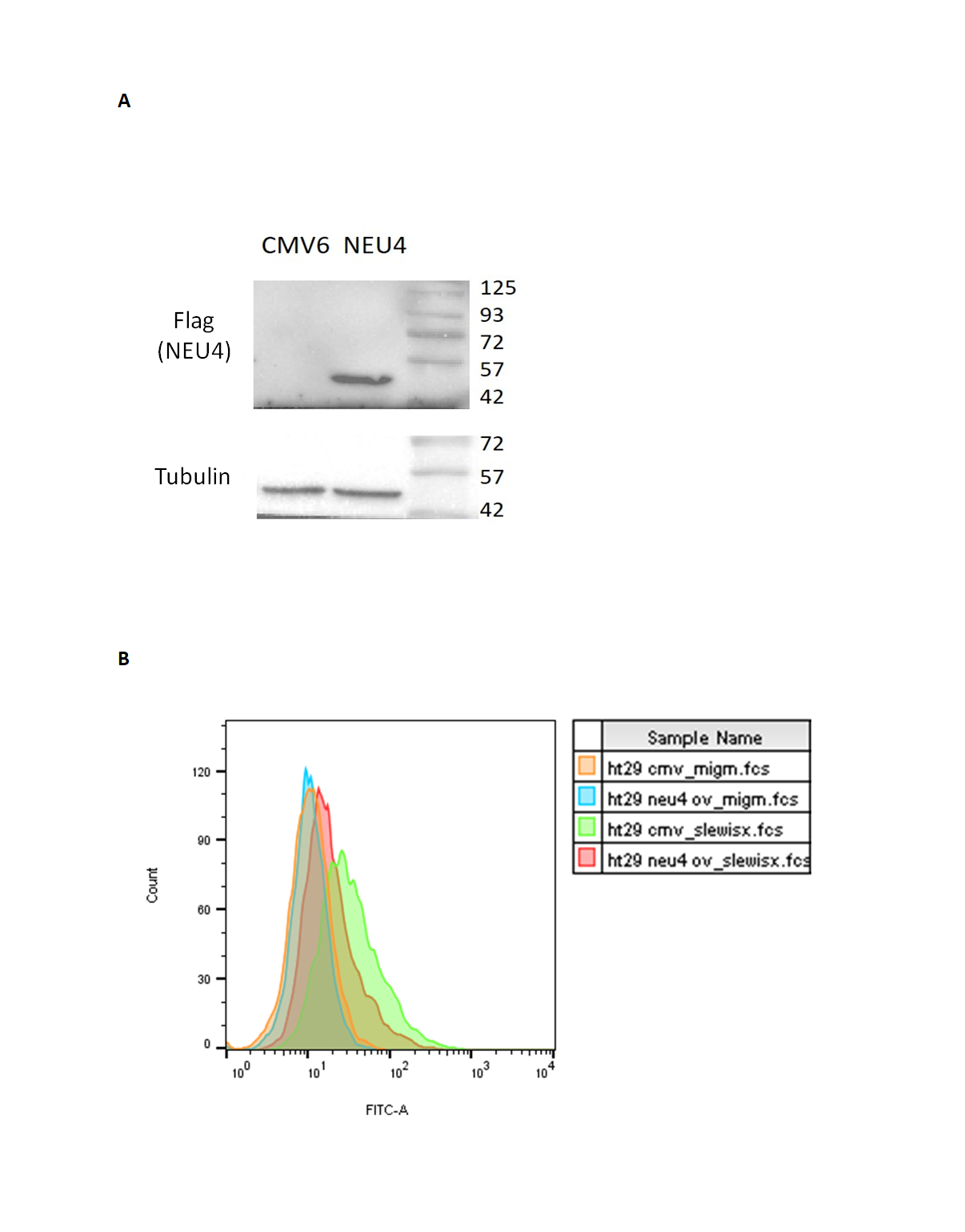
**

**Fig S5. Over-expression of NEU4 represses sialyl Lewis X expression.** (A) over-expression of Flag-tagged NEU4 detected by anti-Flag in HT29 cells. Numbers to the right indicate the molecular weight marker (kDa). CMV6 is the empty vector control. The original full-length blots are shown in supplementary Figure S10. (B) Over-expression of NEU4 repressed sialyl Lewis X expression in HT29 cells (orange line, isotype control with cells transfected with empty vector; blue line, isotype control with cells transfected with Flag-NEU4; green line, sialyl Lewis X staining with cells transfected with empty vector; red line, sialyl Lewis X staining with cells transfected with Flag-NEU4).

**
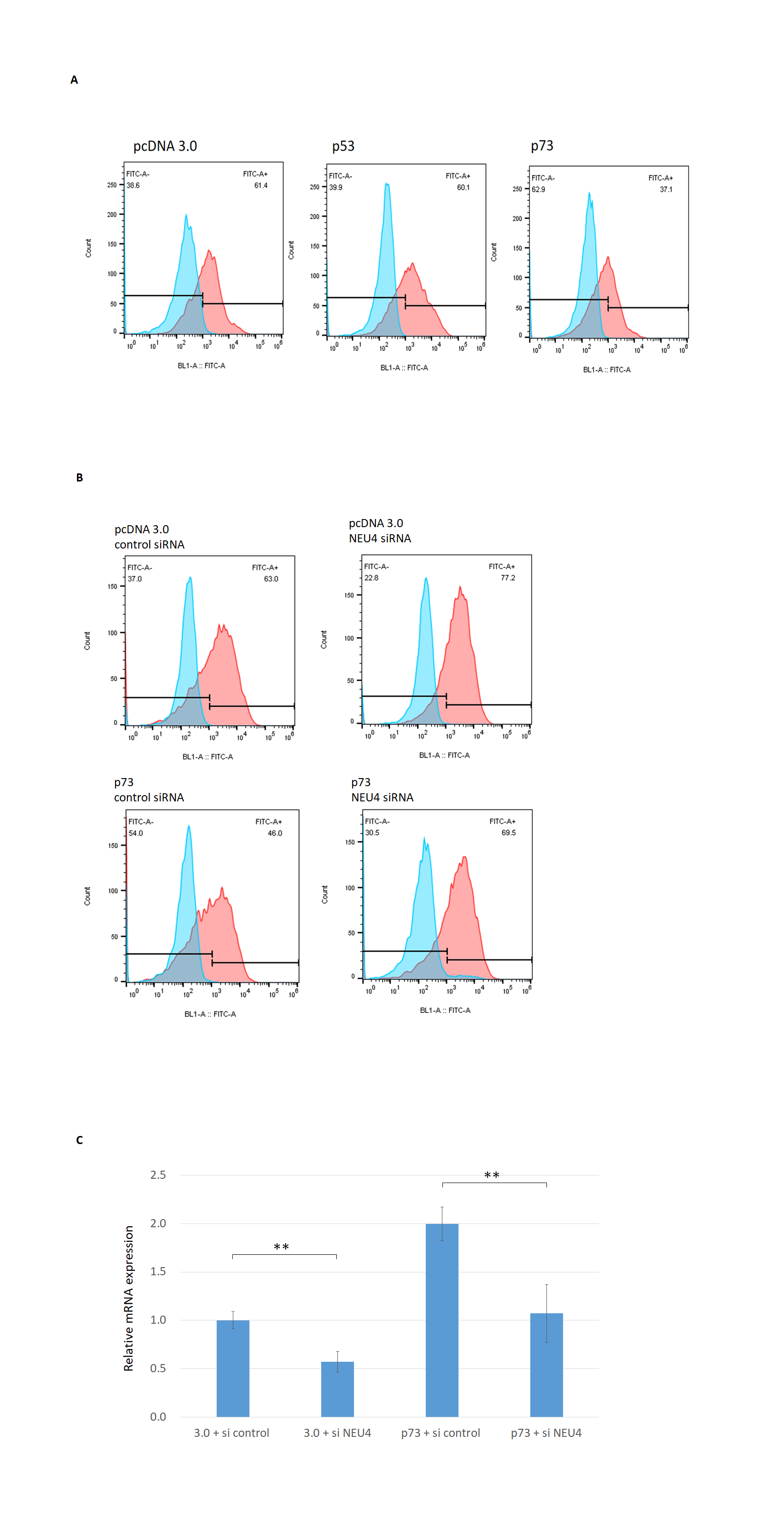
**

**
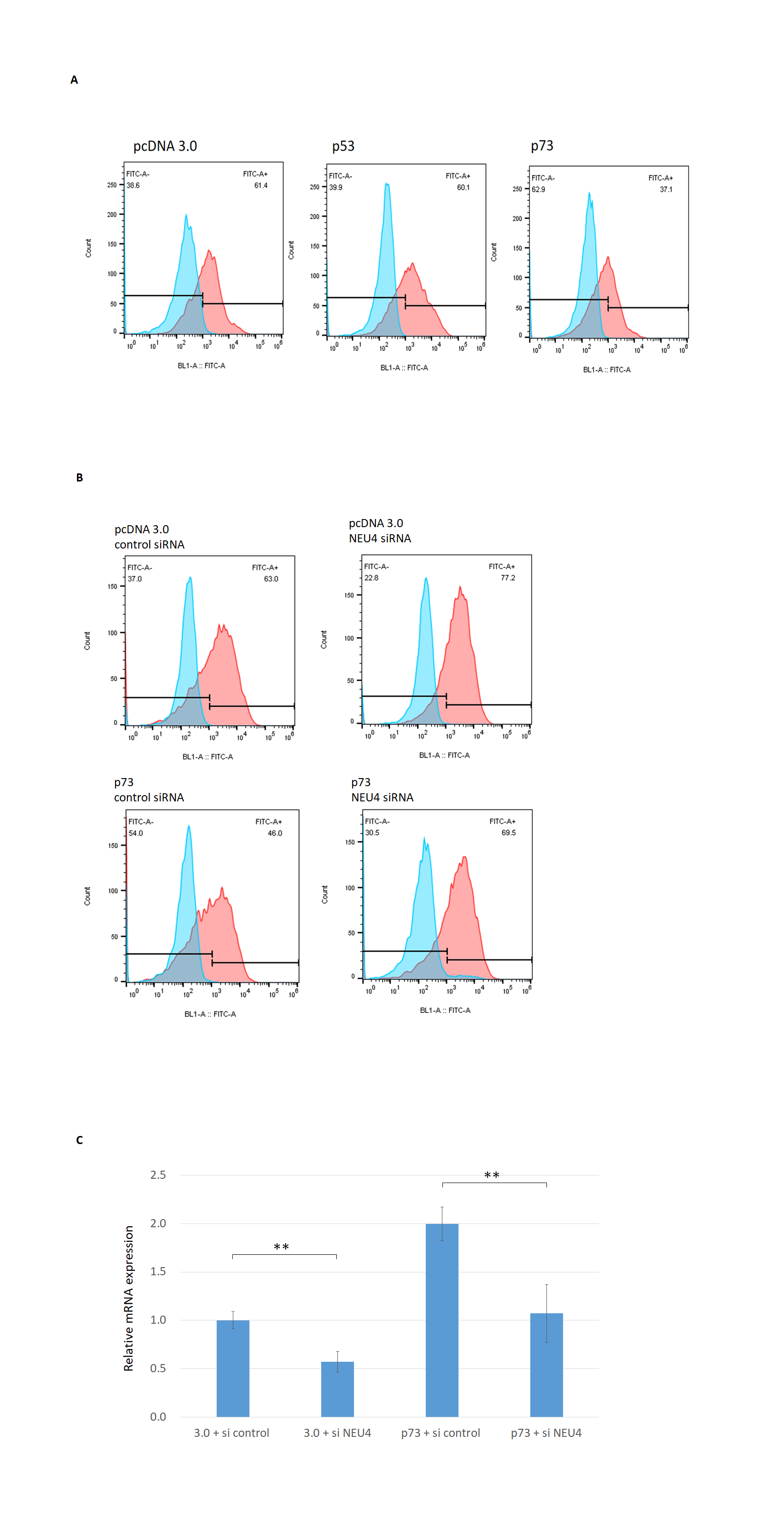
**

**Fig S6. p73, but not p53, can repress sialyl Lewis X expression in LS174T cells. (A)** LS174T cells were transfected with pcDNA3.0 (empty vector) or with p53- or p73- containing vectors and then were assessed for sialyl Lewis X expression by flow-cytometry. p73, but not p53, over-expression repressed sialyl Lewis X expression. The blue line shows the IgM isotype control, and the red line shows sialyl Lewis X staining. (B) LS174T cells were co-transfected with control siRNA or NEU4 siRNA with pcDNA3.0 or p73-vector, and then were assessed for sialyl Lewis X expression by flow-cytometry. Over-expression of p73 repressed sialyl Lewis X expression, but the sialyl Lewis X repression function of p73 were depleted after introduction of NEU4 siRNA. The blue line shows the IgM isotype control, and the red line shows sialyl Lewis X staining. (C) LS174T cells were co-transfected with pcDNA3.0 or p73-vector with control siRNA or NEU4 siRNA, and then were assessed for NEU4 expression. Over-expression of p73 increased NEU4 expression, but the NEU4 enhancement function of p73 were reversed after introduction of NEU4 siRNA. Results are displayed as mean ± SD, n=3 (**p < 0.01).


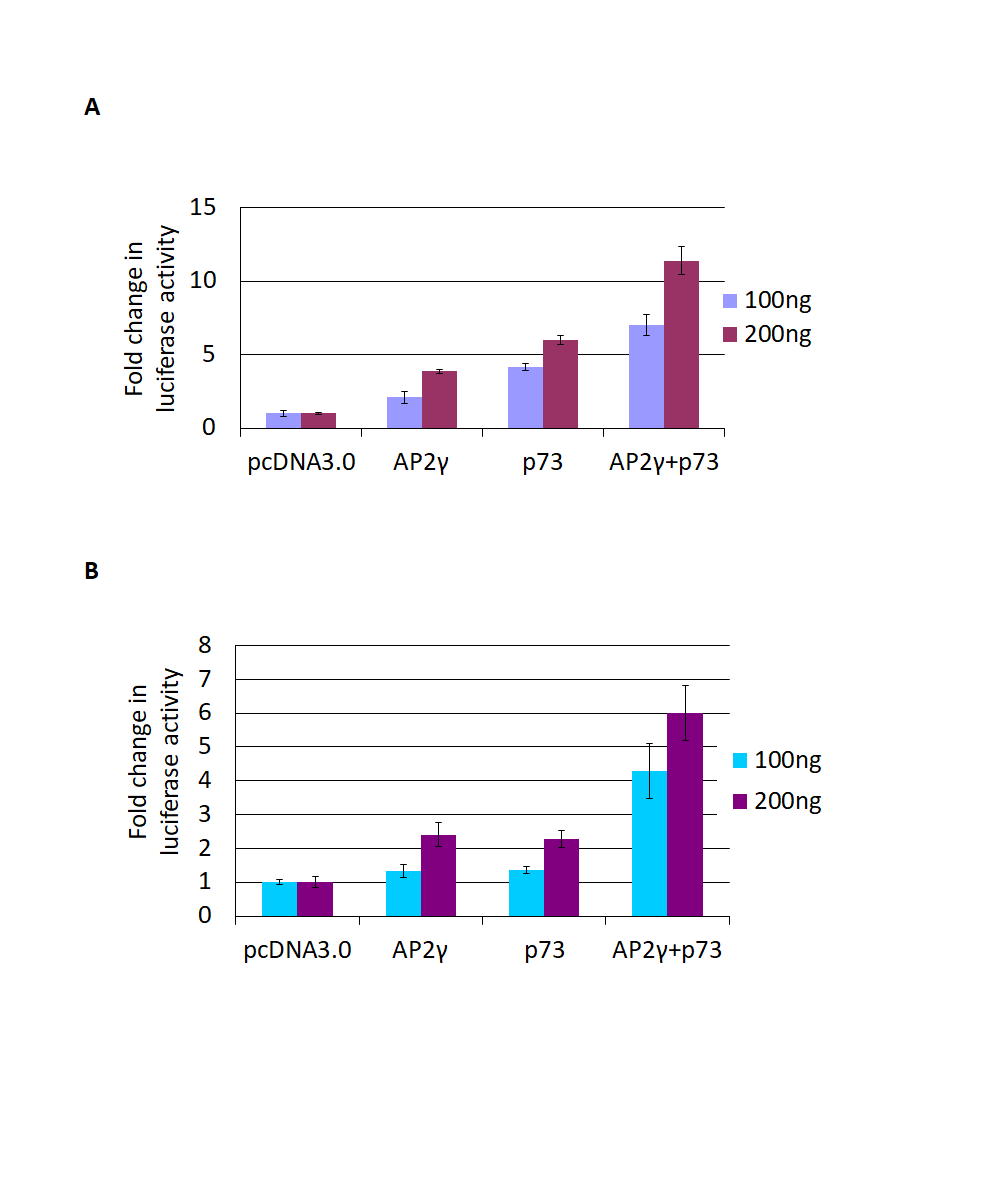


**Fig S7. p73 and AP2γ synergistically activate the NEU4 promoter.** (A) Co-expression of AP2γ and p73 had a much higher activation effect on NEU4 than AP2γ or p73 alone in HT29 cells. (B) Co-expression of AP2γ and p73 had a much higher activation effect on NEU4 than AP2γ or p73 alone in LS174T cells. (A-B) Results are displayed as mean ± SD, n=3


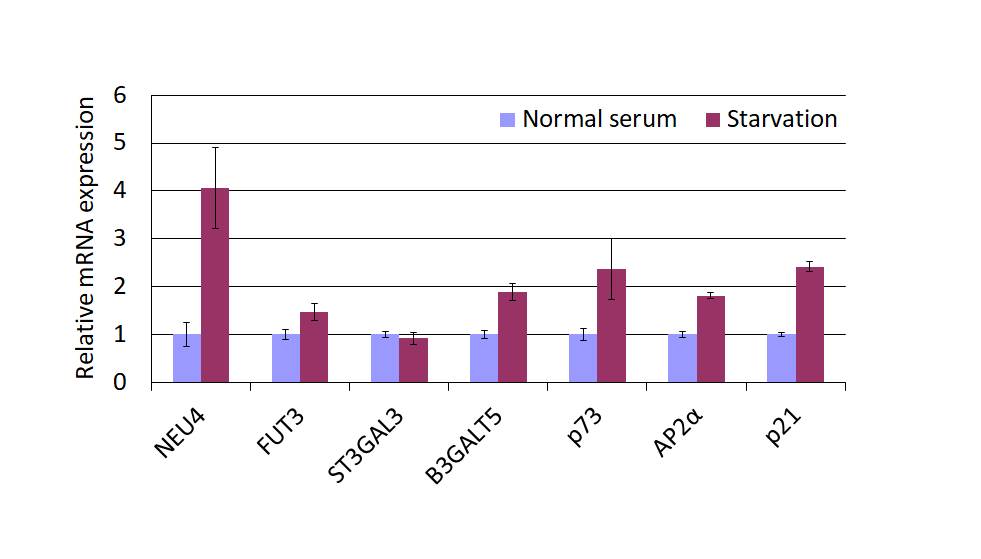


**Fig S8. Gene expression profiles under normal serum conditions and starvation conditions.** The genes of two of the synthetic enzymes for sialyl Lewis A, FUT3 and B3GALT5, were up-regulated upon starvation in HT29 cells. p73, AP2α, P21 and NEU4 were also up-regulated upon starvation. Results are displayed as mean ± SD, n=3

**Fig S**
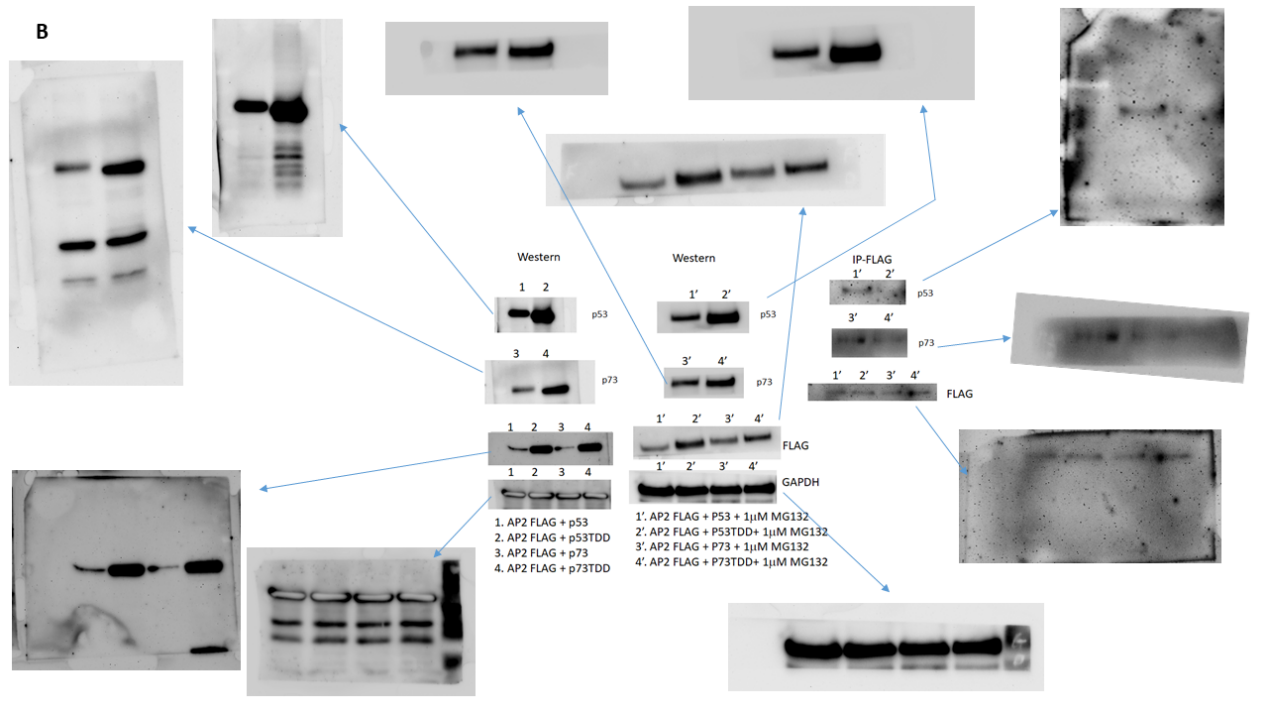

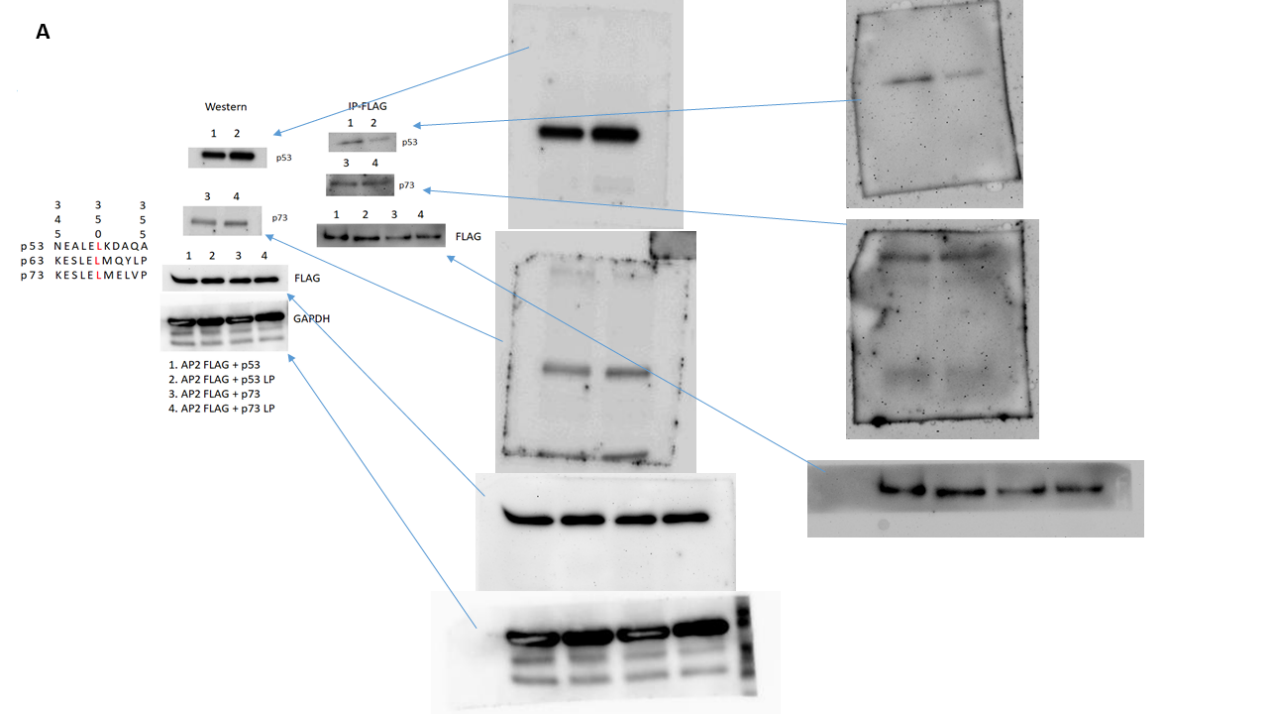
**9. Full-length blots for Figure 7.** (A) The original full-length blots for Figure 7B. (B) Full-length blots for Figure 7C.

**Fig. S**
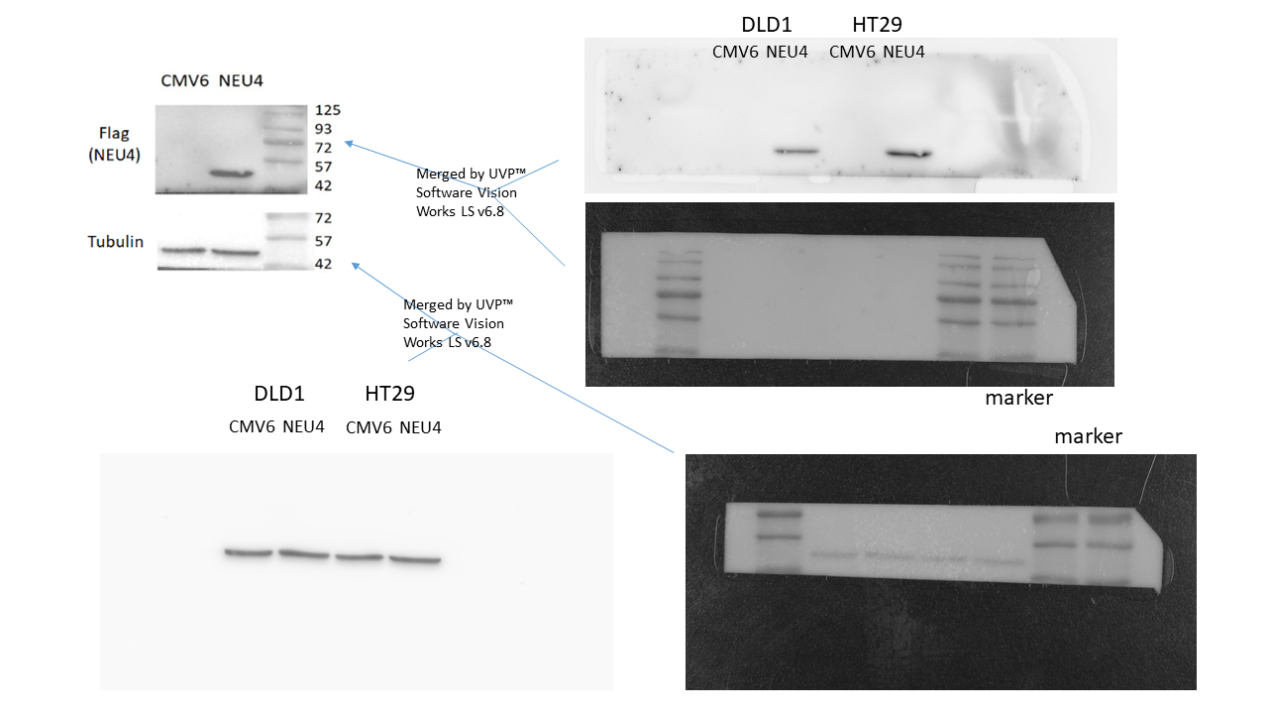
**10. Original full-length blots for Figure S5A.** Over-expression of Flag-tagged NEU4 detected by anti-Flag in DLD1 and HT29 cells. CMV6 is the empty vector control. Blots of Western and marker are merged by UVP BioSpectrum 500 Imaging System VisionWorksLS 6.8.

**Table S1. Primers used for conventional RT-PCR.**

| Gene | Primer sequence (5′−3′) | Length, bp | Annealing, °C |
| --- | --- | --- | --- |
| GAPDH | F: GTCTCCTCTGACTTCAACAGCG | 131 | 60 |
|  | R: ACCACCCTGTTGCTGTAGCCAA |  | 60 |
| NEU1 | F: AAGGCTGAGAACGACTTCGG | 151 | 60 |
|  | R: CCTCAGCAAAGGCGAGAAGA |  | 60 |
| NEU2 | F: CAGAGGAGACTACGACGCAC | 112 | 60 |
|  | R: GTCATACAAGGGGCATGGGT |  | 60 |
| NEU3 | F: TCCCAGCCCTGCTCTACATA | 166 | 60 |
|  | R: AGTGTGGCTTCCATCAGTGG |  | 60 |
| NEU4 | F: GCCTGAGGCCGTGCAGATCG | 160 | 60 |
|  | R: GCCGGGACCCACAGCGAATG |  | 60 |
| NEU4V3 | F: CAGGTCGTCTCTGGGAGGAG | 186 | 60 |
|  | R: GACCCCCATGCTCAGTTTCA |  | 60 |
| NEU4V4 | F: CGGAACGCAGGTCGTCTC | 192 | 60 |
|  | R: CCCATGCTCTGCAGTTTCAGT |  | 60 |
| NEU4V5 | F: GTCCTGACGTAGCCCTGAGAC | 198 | 60 |
|  | R: CACCATGCAGGAGCGGTCA |  | 60 |
| TAp73 | F: GCACCACGTTTGAGCACCTCT | 168 | 60 |
|  | R: GCAGATTGAACTGGGCCATGA |  | 60 |
| ΔNp73 | F: CAAACGGCCCGCATGTTCCC | 256 | 60 |
|  | R: TGGTCCATGGTGCTGCTCAGC |  | 60 |
| ΔN’p73 | F: TCGACCTTCCCCAGTCAAGC | 211 | 60 |
|  | R: TGGGACGAGGCATGGATCTG |  | 60 |
| AP2α | F: ACATGCTCCTGGCTACAAAAC | 70 | 60 |
|  | R: AGGGGAGATCGGTCCTGA |  | 60 |
| AP2γ | F: TGTTTTGGGGGACGCCGGAC | 95 | 60 |
|  | R: ATTGCTGCTCCCGTCGTGGC |  | 60 |
| FUT2 | F: GGACCAGGTGAGAGAAGCCATGC | 117 | 60 |
|  | R: GCTAGCCGCTGCTGAACGTGA |  | 60 |
| FUT3 | F: TGTCCAACCCTAAGTCACGC | 101 | 60 |
|  | R: TTCCAGGTGCTGGCAGTTAG |  | 60 |
| p21 | F: ACCATGTGGACCTGTCACTGT | 170 | 60 |
|  | R: TTAGGGCTTCCTCTTGGAGAA |  | 60 |
| B3GALT5 | F: AGGGTGGTCCTGCGTCCACT | 160 | 60 |
|  | R: ACCCTGCAACACAGGCTCGC |  | 60 |
| ST3GAL3 | F: AATCTGCTGCTAGCCCTCTG | 95 | 60 |
|  | R: TTGGAGTCCTCCTCCCACTG |  | 60 |

**Table S2. Primers used for ChIP assays.**

| Gene | Primer sequence (5′−3′) | Length, bp | Annealing, °C |
| --- | --- | --- | --- |
| NEU4 p73 site | F: TAAATGGGCCCAGGTGTTTAC | 193 | 60 |
|  | R: CCATCAAGAGTTTTGCCCAG |  | 60 |
| NEU4 AP2 site | F: GGTCTGGTCCTGAAGATCTTCC | 202 | 60 |
|  | R: ATGACGAGACCAGGACCCGT |  | 60 |
| NEU4 ORF | F: CCTCAGCTTCACAGTCACCG | 94 | 60 |
|  | R: CTCAGTTTCAGTTCCCGCCA |  | 60 |
| p21 p53 site | F: GTGGCTCTGATTGGCTTTCTG | 105 | 60 |
|  | R: CTGAAAACAGGCAGCCCAAG |  | 60 |
